# Remote sensing for field pea yield estimation: A study of multi-scale data fusion approaches in phenomics

**DOI:** 10.3389/fpls.2023.1111575

**Published:** 2023-03-03

**Authors:** Afef Marzougui, Rebecca J. McGee, Stephen Van Vleet, Sindhuja Sankaran

**Affiliations:** ^1^ Department of Biological Systems Engineering, Washington State University, Pullman, WA, United States; ^2^ United States Department of Agriculture-Agricultural Research Service (USDA-ARS), Grain Legume Genetics and Physiology Research Unit, Washington State University, Pullman, WA, United States; ^3^ Agriculture and Natural Resources, Washington State University Extension, Colfax, WA, United States

**Keywords:** high-resolution satellite, unmanned aerial system, multispectral, yield prediction, pan-sharpening, high-throughput field phenotyping, plant breeding

## Abstract

**Introduction:**

Remote sensing using unmanned aerial systems (UAS) are prevalent for phenomics and precision agricultural applications. The high-resolution data for these applications can provide useful spectral characteristics of crops associated with performance traits such as seed yield. With the recent availability of high-resolution satellite imagery, there has been growing interest in using this technology for plot-scale remote sensing applications, particularly those related to breeding programs. This study compared the features extracted from high-resolution satellite and UAS multispectral imagery (visible and near-infrared) to predict the seed yield from two diverse plot-scale field pea yield trials (advanced breeding and variety testing) using the random forest model.

**Methods:**

The multi-modal (spectral and textural features) and multi-scale (satellite and UAS) data fusion approaches were evaluated to improve seed yield prediction accuracy across trials and time points. These approaches included both image fusion, such as pan-sharpening of satellite imagery with UAS imagery using intensity-hue-saturation transformation and additive wavelet luminance proportional approaches, and feature fusion, which involved integrating extracted spectral features. In addition, we also compared the image fusion approach to high-definition satellite data with a resolution of 0.15 m/pixel. The effectiveness of each approach was evaluated with data at both individual and combined time points.

**Results and discussion:**

The major findings can be summarized as follows: (1) the inclusion of the texture features did not improve the model performance, (2) the performance of the model using spectral features from satellite imagery at its original resolution can provide similar results as UAS imagery, with variation depending on the field pea yield trial under study and the growth stage, (3) the model performance improved after applying multi-scale, multiple time point feature fusion, (4) the features extracted from the pan-sharpened satellite imagery using intensity-hue-saturation transformation (image fusion) showed better model performance than those with original satellite imagery or high definition imagery, and (5) the green normalized difference vegetation index and transformed triangular vegetation index were identified as key features contributing to high model performance across trials and time points. These findings demonstrate the potential of high-resolution satellite imagery and data fusion approaches for plot-scale phenomics applications.

## 1 Introduction

Crop improvement efforts focus on developing new cultivars with increased yield potential, stable agronomic traits, and better environmental adaptability. Genomic tools and technologies have played a major role in advancing plant breeding programs by enabling “accurate” quantitative trait loci mapping and genome-wide association studies (Varshney et al., 2021). However, locating associated genes depends on the accuracy of the phenotypic data (Pratap et al., 2019). The acquisition of phenotypic data needs accurate, rapid, and efficient tools to bridge the relationship between the genotype and phenotype and the interactions of the genotype with the environment and management practices. Phenomics or high-throughput plant phenotyping technologies have enabled accurate and rapid phenotyping at different scales and resolutions (Yang et al., 2020).

The rise of field phenomics is driven by the advances in sensing technologies that can be deployed using drones or unmanned aerial systems (UAS) (Sankaran et al., 2015; Pieruschka and Schurr, 2019; Zhao et al., 2019a; Yang et al., 2020; Guo et al., 2021). Such systems have been used for various phenomics applications – from spotting ideotypes (Roth et al., 2022) to studying latent heat flux (Tauro et al., 2022) in crops. The success of UAS-based sensing techniques for measuring crop phenotypes in breeding trials is contributed to the following: (i) low altitude flights enable acquisition of high spatial resolution data (Zhang et al., 2020; Jin et al., 2021), ideal for imaging the small-size plots; (ii) UAS equipment and cameras are small, light, and easily portable (Zhao et al., 2019a; Zhang et al., 2020); (iii) the flexibility of UAS in selecting the date and time for data acquisition (Zhang et al., 2020) or collecting data at different times within a day (Valencia-Ortiz et al., 2021); and (iv) the ability of the system to be integrated with single or multiple on-board sensors such as RGB, multispectral, hyperspectral, thermal, and/or light detection and ranging (LiDAR) sensing systems, which facilitates the measurements of a wide range of crop traits (Yang et al., 2017; Zhao et al., 2019a; Jin et al., 2021).

One of the key performance traits in crop breeding programs is yield and its components. As these traits are complex and influenced by the environment, there is a continuous and ongoing effort to identify novel approaches, including remote sensing applications, to predict yield and yield-associated traits. These applications rely on retrieving image-based features that can be indirectly associated with yield. A large body of literature reports the accuracy of vegetation indices (VIs) in predicting yield and other important agronomic and stress-tolerant traits at breeding plot level for multiple crops such as wheat (Kyratzis et al., 2017; Hassan et al., 2018; Hassan et al., 2019; Li et al., 2019; Fu et al., 2020; Shafiee et al., 2021; Zeng et al., 2021), soybean (Zhang et al., 2019; Maimaitijiang et al., 2020a; Maimaitijiang et al., 2020b; Roth et al., 2022; Santana et al., 2022), maize (Buchaillot et al., 2019; Adak et al., 2021; Sankaran et al., 2021), and pulse crops (Sankaran et al., 2018; Marzougui et al., 2019; Vargas et al., 2019; Valencia-Ortiz et al., 2021; Zhang et al., 2021; Tefera et al., 2022). While yield prediction using UAS-based sensing approaches has shown promising results, scaling up the application to cover large areas and/or multi-environment trials is still a major challenge (Zhang et al., 2020; Jin et al., 2021; Smith et al., 2021). To determine yield stability, crop breeders need to evaluate the breeding plant materials at different geographical locations and assess genotype to environment and management (G x E x M) interactions. In addition, it is important to note that the breeding cycle may require 10 or more years from the first genetic cross to commercial release of new varieties (Tracy et al., 2020; Kholová et al., 2021). This factor strongly depends on the crop type and environments (Watt et al., 2020). For instance, in the United States Department of Agriculture Agricultural Research Services’ (USDA-ARS) pea breeding program in the Pacific Northwest USA, the total number of locations that each breeding plant material is evaluated in a given season can range from 4-15 (**Supplementary Materials Figure 1**) depending on whether it is entered in local, state-wide, or regional trials within the USA. Thus, scaling up the UAS-based phenomics is not only limited by the spatial coverage, and depends on several factors such as personnel, equipment, and travel time. In addition, other limitations could result from the airspace regulations, especially if trials are within a short distance of an airport (Yang et al., 2020; Zhang et al., 2020), limited access to remote locations (Zhang et al., 2020), and finite battery capacity and flight time (Guo et al., 2021). Therefore, to address some of these limitations, in this research, our major focus is towards exploring the potential of low-orbiting high-resolution satellite imagery as a tool and its capacity for multi-scale, multi-sensor (with UAS data) and multi-modal (spectral and texture) data integration to improve the scalability of field phenomics applications.

The availability of low-orbiting high-resolution satellite imagery offers a great opportunity to measure phenotypic traits at the breeding plot scale. Such application was not previously possible due to spatial and temporal resolution constraints. For example, in the Pacific Northwest region of the USA, the size of breeding plots in spring wheat, pea, and chickpea breeding programs is about 1.5 x 6 m (Sangjan et al., 2021; Zhang et al., 2021). Segmenting each plot with coarse resolution imagery data was not possible. Nevertheless, as described in Zhang et al. (2020), there is a potential to leverage the very high-resolution satellite imagery for field phenomics applications, particularly utilizing the available commercial imagery with a spatial resolution of 0.30-0.50 m/pixel. In addition, these sources have a high temporal resolution, and the image acquisition time points can be tasked to the desired time window within a short period (less than a week), as long as cloud cover is not a limitation. The applications are particularly promising for crop breeding programs in the semi-arid agricultural production areas due to the preponderance of cloud-free days.

Combining multi-modal, multi-scale, multi-sensor data from satellite and UAS sources can also offer a more robust solution, especially for agricultural monitoring. Alvarez-Vanhard et al. (2021) reviewed the different scenarios of satellite-UAS information synergies for agricultural and non-agricultural applications and proposed four strategies that will benefit from satellite-UAS integration. These strategies include data comparison, multi-scale explanation, model calibration, and data fusion. In terms of data comparison, Sankaran et al. (2021) demonstrated that satellite-based vegetation indices were significantly correlated with those from UAS imagery and seed yield in a maize breeding trial. Multi-scale explanation in agriculture may include anomaly detection using satellite data, with deeper and precise assessment of underlying anomaly using UAS imagery. In Selvaraj et al. (2020), banana plantations were identified using machine learning approaches applied to satellite imagery (Planet and WorldView2), while UAS data were used to identify the major diseases in the canopy within those satellite imageries identified banana plantations. Examples of model calibration are the integration of satellite (with mixed pixels) and UAS data (with unmixed pixels) to develop a spectral un-mixing approach for improving the mapping of different plant communities (Alvarez-Vanhard et al., 2020), and improving the classification accuracies (irrigated area mapping) of the satellite-based data using the labeled dataset acquired with the UAS imagery (Nhamo et al., 2018). For the data fusion, both image and feature-based fusion can be explored. For example, Malamiri et al. (2021) enhanced the accuracy in classifying six pistachio cultivars (with precise weed and soil separation) using pan-sharpened Landsat 8 imagery with a UAS RGB image, which increased the spatial resolution of satellite imagery to 20 cm/pixel. Similarly, Zhao et al. (2019b) fused UAS RGB images with Sentinel-2A to improve the accuracy of crop mapping with an enhanced spatial resolution of 10 cm/pixel. Maimaitijiang et al. (2020a) found that the combination of canopy structure features extracted from UAS RGB data and canopy spectral features extracted from satellite multispectral data improved the estimation of soybean above ground biomass, leaf area index, and leaf nitrogen content compared to just a single sensor approach. Even if such studies in the literature that investigate the integration of satellite and UAS data are increasing, the studies exploring such synergies in the context of field phenomics involving small plot applications, especially in field pea, with low-orbiting high-resolution satellite imagery are limited.

Therefore, to help bridge this gap, in this study, we evaluated multiple approaches involving the integration of UAS and high-resolution satellite imagery to predict seed yield of field pea entries in breeding trials. We hypothesize that: (i) the quality of satellite-based features will be comparable to UAS-based features (to determine the suitability of satellite imagery for phenotyping applications in small plot research), (ii) the feature fusion from both sensing approaches will capture the temporal change of crop growth, and (iii) the fusion of satellite and UAS imagery will improve satellite image spatial resolution and thus the accuracy of the extracted features. The aspects mentioned above were evaluated by developing machine learning models for seed yield prediction and assessing information gains utilizing data from multiple sensors (versus a single sensor), and single and multiple time points. We explored the integration of satellite and UAS vegetation metrics extracted from multispectral imagery to enhance the seed yield estimation at both feature and image levels.

## 2 Materials, data acquisition, and pre-processing

### 2.1 Study site and experimental design

**Figure 1:**
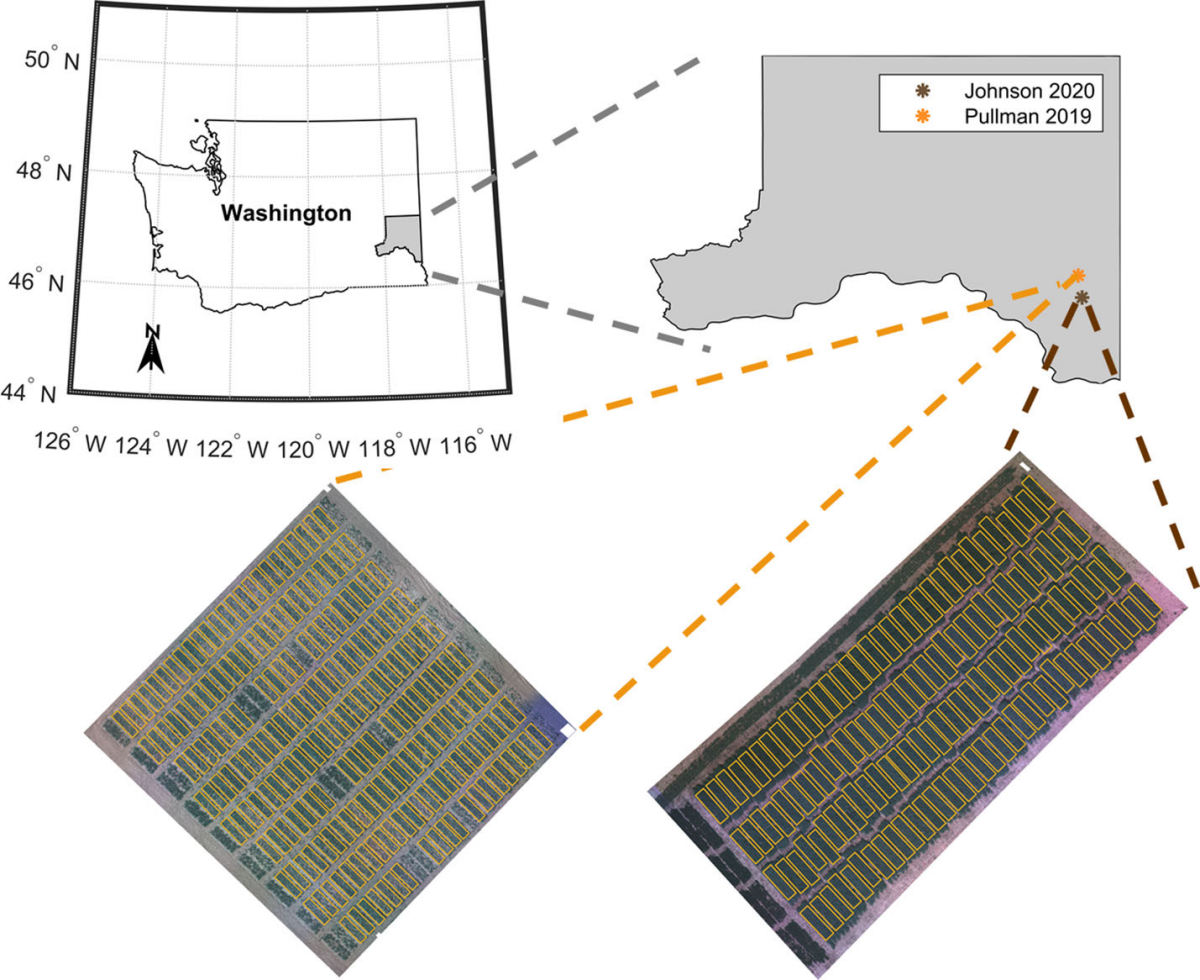
Location of the pea breeding field trials located in the Pacific Northwest region of USA in the state of Washington, USA: advanced yield trial grown in Pullman, Washington (including check varieties used as reference) evaluated in 2019 season (Site 1) and variety test trial grown in Johnson, Washington (including check varieties used as reference) evaluated in 2020 season (Site 2).

This study evaluated two field pea-breeding trials in two consecutive years (**Figure 1**). The advanced breeding trial, which we denote as ‘Site 1’ hereafter in this text, refers to the USDA-ARS replicated advanced yield trial at the end of the crop breeding cycle, prior to releasing a new cultivar. This trial was evaluated in 2019 near Pullman, Washington, USA. The variety testing trial (termed ‘Site 2’) was evaluated in 2020 at Johnson, Washington, USA. This trial comprises plant materials from both private and public breeding programs, including some commercial varieties. Both trials were planted using a randomized complete block design with three and four replications for Site 1 and Site 2, respectively. The advanced yield trial at Site 1 contained three adjacent trials – one in which all entries had green seeds, one in which all entries had yellow seeds, and one in which the entries had either yellow or green seeds. There were 65 entries total. The Site 2 trial included 33 entries with a 24% overlap with the Site 1 trial (8 common entries). The entries had either green or yellow seeds. In this trial, all seeds were inoculated with *Rhizobium* bacteria before sowing. For both trials, seeds of each entry were planted in separate plots. Each plot was 6.1 m long and 1.5 m width and had 6 rows. There was 0.75 m between plots.

### 2.2 Ground reference data

The seed yield (kg/ha) was collected from each plot at physiological maturity in August 2019 and 2020. The seed yield for both trials (Site 1 and Site 2) had a normal distribution (**Supplementary Materials Figure 2**). The summary statistics of seed yield collected across the two years are presented in **Table 1**. Plots without yield data points and plots recognized as outliers (low yield) were excluded from further analysis. Moreover, some check varieties had more than three or four replicates. The 2020 field season was a longer season, with favorable weather conditions resulting in high yields and lower yield variance than the 2019 field season.

**Table 1:** Summary statistics of field pea seed yield (kg/ha) from two locations.

Trials	No. of entries	No. of plots	Median	Mean	Max	Min	SD	CV (%)
Site 1	65	203	1964	1938	3195	662	498	26
Site 2	33	131	4476	4514	6395	2442	783	17

Site 1, advanced yield trial; Site 2, variety testing trial; SD, standard deviation; CV, coefficient of variation. One plot with low yield was considered as outlier and was removed from each trial.

### 2.3 Satellite and UAS data acquisition

**Figure 2:**
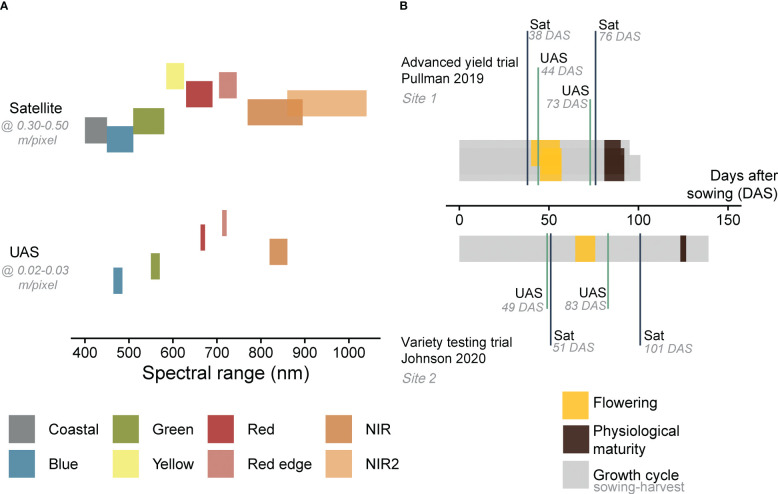
Spectral range of satellite and UAS multispectral imagery acquired in this study **(A)**. The data acquisition time points of remote sensing data **(B)**. Details about spectral resolution and acquisition dates are summarized in **Supplementary Materials Table 1**. DAS refers to days after sowing.

Multispectral images were collected using a quadcopter UAS (AgBot, ATI Inc., Oregon City, Oregon, USA), equipped with a five-band multispectral camera (RedEdge MX, Micasense Inc., Seattle, Washington, USA; **Figure 2A** and **Supplementary Materials Tables 1, 2**). Images were acquired with a resolution of 1.2 MP and dynamic range of 12-bit, at flying altitude of 25 m in 2019 (ground sampling distance (GSD) = 0.02 m/pixel) and 30 m in 2020 (GSD = 0.03 m/pixel). The flying speed was set to 2.5 m/s and the forward and side overlap to 80% in 2019 and 70% in 2020. Images covering the area of interest were stitched to generate an orthomosaic using Pix4Dmapper (Pix4D Inc., Lausanne, Switzerland). The description of the UAS image pre-processing technique can be found in Zhang et al. (2021). The UAS data collection was conducted twice at each season (17 June 2019 and 16 July 2019 at Site 1, 02 June 2020 and 06 July 2020 at Site 2). These time points coincided with two different growth stages of field pea (flowering and pod development at Site 1, vegetative stage and pod development at Site 2, **Figure 2B**). The orthomosaic bands [5 bands x 2 time points (TP 1 and TP 2) x 2 locations (Site 1 and Site 2)] were radiometrically calibrated by converting the raw imagery values (digital numbers) to surface reflectance using a Spectralon reflectance panel (99% reflectance; Spectralon, SRS-99-120, Labsphere Inc., North Sutton, NH, USA).

Satellite imagery from WorldView (-2 or -3) were obtained at the closest time to the UAS acquisition dates (11 June 2019 and 19 July 2019 at Site 1, 04 June 2020 and 24 July 2020 at Site 2, **Figure 2B** and **Supplementary Materials Tables 1, 2**). The satellite revisit frequency at a specific location is determined by factors such as the satellite’s altitude, orbit, desired coverage area and location, as well as weather conditions. On average, the revisit time for WorldView satellites is approximately one day at 1 m GSD. The data were delivered by Maxar Technologies (Westminster, Colorado, USA) as standard level 2A with atmospheric compensation, which accounts for atmospheric scattering effects on the data. The imagery products were delivered as surface reflectance and comprised eight spectral bands in the visible near-infrared region (**Figure 2A**). The spatial resolution of the eight spectral bands in WorldView-2 or -3 ranges from ~1.8 to 1.2 m, while the panchromatic band has a resolution of ~ 0.50 or 0.30 m, respectively, depending on the sensor. The high-resolution panchromatic bands were then combined with the lower resolution multispectral bands to create a pan-sharpened image with improved spatial resolution and the final products were acquired with a GSD of about 0.30-0.50 m/pixel. In this study, the coastal bands were not included, resulting in [7 bands x 2 time points (TP 1 and TP 2) x 2 locations (Site 1 and Site 2)].

The images from both satellite and UAS sources were co-registered and aligned using the Georeferencer tool in QGIS (QGIS.org, 2021, version 3.10.16). The images were then cropped to the same region of interest covering the breeding trials. At each site, two shapefiles were manually created using the Digitizing tool in QGIS to delineate the boundaries of each plot. The alignment of satellite and UAS imagery facilitated the segmentation of plot boundaries.

## 3 Data processing and analysis

### 3.1 Overview of data analysis

**Figure 3:**
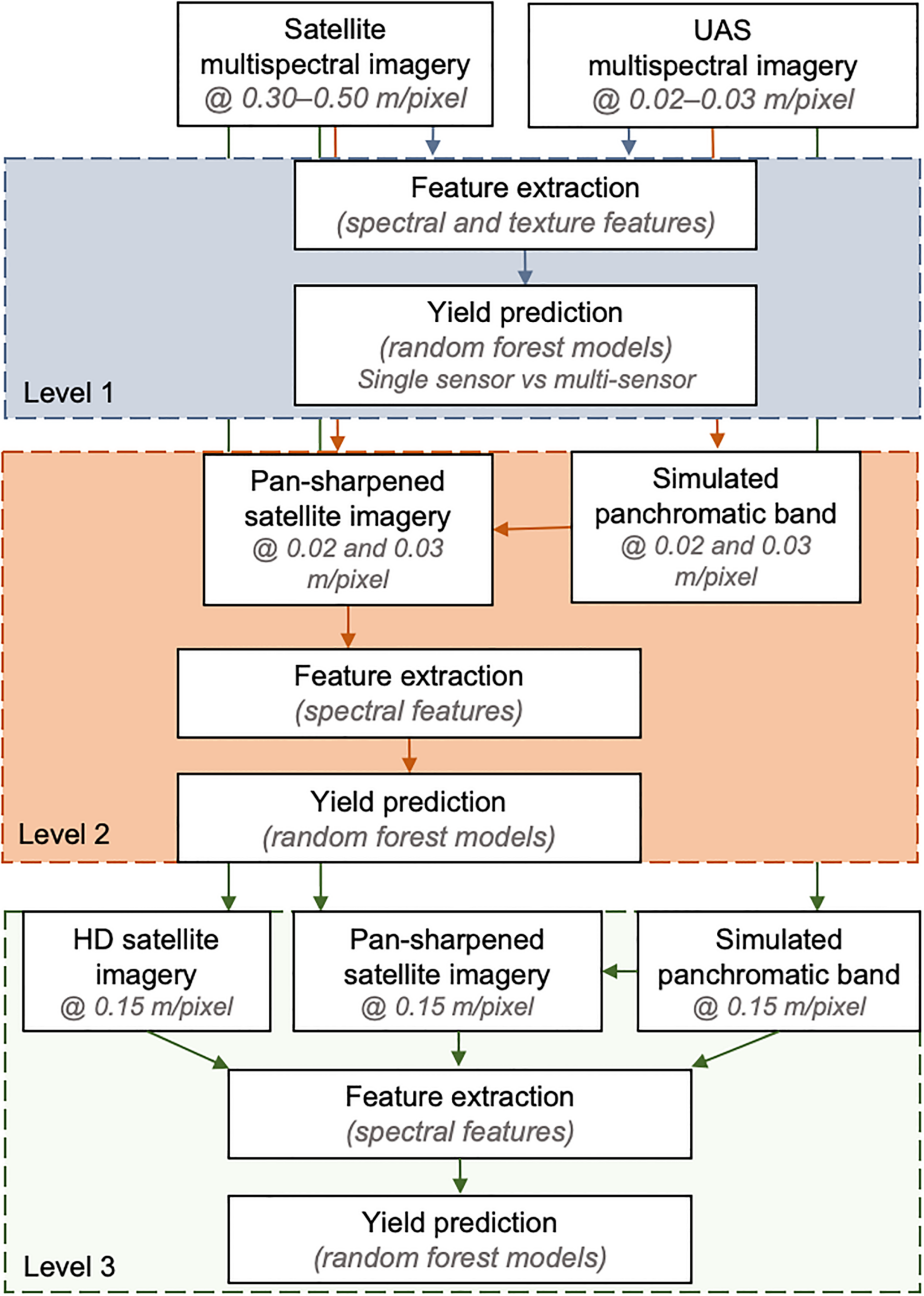
Image processing workflow for analyzing data extracted from satellite and UAS imagery. The top, middle and lower boxes refer to the evaluations in Level 1, Level 2, and Level 3, respectively. Level 1, 2, and 3 included feature extraction and yield prediction using extracted features from original resolution of the satellite and UAS imagery, pan-sharpening of satellite imagery using UAS imagery and feature extraction from the pan-sharpened imagery for yield prediction, and comparison of yield prediction using pan-sharpened image-based features and HD image features, respectively.

The study explored the impact of different spatial resolutions and data fusion techniques on field pea seed yield estimation using remote sensing data. The analysis workflow was structured into three levels, as depicted in **Figure 3**.

The first level (Level 1) involved utilizing the surface reflectance imagery at its original spatial resolution. The GSD of satellite imagery was 0.30-0.50 m/pixel and the UAS imagery had a GSD of 0.02-0.03 m/pixel. The multispectral imagery was further processed to extract features that describe the spectral and texture information. Using these features as input, random forest models were trained to predict field pea seed yield. The performance of the models was evaluated using features extracted from multi-scale data (satellite and UAS), as well as features from single- and multi-time points. The assessment of the models incorporating features from multi-time points refers to the fusion of multi-modal (spectral and textural) and multi-scale vegetation features, extracted from both satellite and UAS imagery at combined time points.

The second level (Level 2) focused on evaluating the impact of image fusion on field pea seed yield estimation. This was achieved by enhancing the spatial resolution of satellite imagery using two pan-sharpening techniques with UAS data to reach a satellite image GSD of 0.02-0.03 m/pixel. The panchromatic bands were simulated by averaging the five UAS spectral bands. The quality of the resulting synthesized pan-sharpened satellite imagery was evaluated using five image evaluation metrics, which are described in more detail in the next section. Additionally, the spectral quality of the pan-sharpened imagery was assessed by training random forest models to predict seed yield using spectral features (vegetation indices) derived from these pan-sharpened satellite imagery. These evaluations were conducted for both Site 1 and Site 2 data.

The final analysis level (Level 3) focused on comparing between two techniques of satellite image spatial resolution enhancement for seed yield estimation. The two techniques for enhancing satellite image spatial resolution were super-resolution and pan-sharpening. The super-resolution images were acquired as “High-Definition” products (HD) with a GSD of 0.15 m/pixel from Maxar Technologies. The HD products were generated using their proprietary super-resolution technique, which enhances the spatial details of the satellite image during post-processing using a machine learning approach. Since the HD satellite imagery was only available for Site 2 (two–time points), this aspect was assessed only for this location. These images were compared to the pan-sharpened satellite imagery (similar to those described in Level 2) but with a spatial resolution of 0.15 m/pixel and four spectral bands – RGB and NIR to match the HD image spatial resolution.

### 3.2 Satellite pan-sharpening

Image fusion is the process of combining multiple images taken at different times or with different sensors to create a single image that contains more information than the original imagery. Pan-sharpening is a type of image fusion approach, which combines the high spatial resolution of the panchromatic band (having a high spatial resolution) with a lower resolution multispectral imagery (RGB or multispectral images) to enhance the spatial resolution of the latter. The result is a single image that with usually a high spatial and spectral resolution. This technique is commonly used to enhance the quality and resolution of the satellite imagery. There are multiple approaches that have been developed for image fusion, each with varied performance efficiencies (Ehlers et al., 2010; Yokoya et al., 2017; Gašparović et al., 2019; Ghamisi et al., 2019; Dadrass Javan et al., 2021). In general, image fusion can be broadly characterized into component substitution, multi-resolution analysis, variational optimization-based techniques, and machine learning-based approaches (Vivone et al., 2019; Vivone et al., 2021). This study selected a technique representing the component substitution and the multi-resolution approaches. These techniques were adopted from the MATLAB Pan Sharpening toolbox (Vivone et al., 2015).

The intensity-hue-saturation (IHS) transformation is a component substitution approach, where the bands of the low-resolution multispectral imagery are converted to IHS components. The intensity component is replaced by the panchromatic band after histogram matching (Al-Wassai et al., 2011; Johnson et al., 2014). In general, the extraction of the intensity component is performed by averaging the bands in the visible region (red, green, blue). In this study, we computed the intensity component by averaging all the spectral bands from the low-resolution satellite imagery (Yilmaz et al., 2019), excluding the coastal band. Similarly, additive wavelet luminance proportional (AWLP) pan-sharpening, which is a multi-resolution analysis approach, was utilized for image spatial enhancement. In the AWLP approach, a low-spatial resolution image is decomposed into scale levels while injecting the panchromatic band matched by each decomposed layer and applying an inverse transformation (Dadrass Javan et al., 2021).

The ideal pan-sharpened imagery should have the same spatial properties as the high-resolution panchromatic band and the same spectral properties as the multispectral input bands, though the process can lead to spectral and/or spatial distortions (Siok et al., 2020). The assessment of the quality of the resulting pan-sharpened imagery can be conducted using Wald’s protocol, which states that the pan-sharpening process is reversible, and that the original multispectral imagery can be obtained by degrading the pan-sharpened imagery (Wald et al., 1997; Vivone et al., 2015). In this study, the spectral and spatial qualities of the pan-sharpened imagery were assessed using five statistical metrics (correlation coefficient CC, structural similarity index measure SSIM, spectral angle mapper SAM, erreur relative globale adimensionnelle de synthese ERGAS, and peak signal to noise ratio PSNR), after degrading their spatial resolution to match that of the original satellite multispectral imagery (Borra-Serrano et al., 2015; Luo et al., 2018; Li et al., 2020; Siok et al., 2020).

### 3.3 Background removal and feature extraction

Background segmentation and soil removal were conducted prior to feature extraction. A threshold based on the soil adjusted vegetation index (SAVI) pixel values was utilized for UAS imagery. However, for satellite imagery, segmentation was based on histogram distribution of SAVI pixel intensity. Thresholds were set as 15% for satellite imagery at spatial resolution of 0.15, 0.30, and 0.50 m/pixel and 25% for the pan-sharpened satellite imagery at 0.02 and 0.03 m/pixel. These thresholds were selected based on visual observations of the SAVI intensity distribution and implemented to eliminate spectrally mixed pixels. Median reflectance values from each vegetation index (normalized difference vegetation index NDVI, green normalized difference vegetation index GNDVI, normalized difference red-edge index NDREI, soil adjusted vegetation index SAVI, atmospherically resistant vegetation index ARVI, transformed triangular vegetation index TVI, infrared percentage vegetation index IPVI, renormalized difference vegetation index RDVI, two-band enhanced vegetation index EVI2, normalized difference red-edge index – with yellow band NDRE2, and normalized difference vegetation index – with NIR2 band NDVI2) were extracted as canopy spectral features. Additionally, texture features were extracted from the individual spectral bands (5 bands for UAS data and 7 bands for satellite data) using the GLCM (grey level co-occurrence matrix) approach. These features included contrast (CO), homogeneity (HO), correlation (CR), and energy (EN). A detailed summary of extracted features is provided in **Supplementary Materials Table 3**. All image-related analyses were conducted using a customized script in MATLAB (Matlab, 2021b; MathWorks Inc., Natick, Massachusetts, USA).

### 3.4 Statistical analysis, yield prediction, and feature importance

The statistical analysis, model development and validation, feature assessment, and visualization were performed in R (http://www.r-project.org/; release 4.0.5). The correlation analysis was performed to evaluate the similarity between the extracted imagery-based features and harvested seed yield at each time point and for each type of imagery data (satellite and UAS). Moreover, machine learning algorithms were applied to estimate harvested seed yield and evaluate the importance of the extracted features. Random forest models were constructed using *randomForest* implementation in caret (Kuhn, 2008). The models were trained using non-scaled data. The coefficients of determination (R^2^) and root mean square error (RMSE) were computed to evaluate the performance of the yield prediction model. The mean ± standard deviation computed from multiple runs was reported for both R^2^ and RMSE.

Each type of data was divided into training and testing sets (80/20). At the beginning of our analysis, two training frameworks were tested: random holdout plot and random holdout entry. For the random holdout plot (plots were held back for testing irrespective of entry), 80% of the data were randomly chosen. For the random holdout entry (all replicates from an entry were held back for testing), 80% of the entries were randomly chosen. The models trained with random holdout entry showed stable performance with lower variation in the testing set than those trained with random holdout plot (**Supplementary Materials Tables 4, 5**). Therefore, we selected the random holdout entry as a data resampling technique.

The random forest hyperparameters were kept at the default level, and only the number of variables at each split was tuned using 3-fold internal cross-validation repeated 15 times. The data split was repeated 10 times by setting 10 random seeds in each run (referred to as 10 random runs henceforth) to assess the variability of model performance with different data splits. The input spectral features used to train the models are summarized in **Supplementary Materials Table 6**. In addition to spectral features extracted from individual sensor UAS and satellite at original spatial resolution at each time point and combined time points, integrating spectral and texture features was also evaluated. Before training the model for each random run, feature selection was conducted on the training dataset to remove highly correlated features with a correlation coefficient threshold of 0.99.

Feature importance was evaluated using a permutation-based method (increased mean squared error IncMSE after removing one feature). Three ranks were created based on three factors to create a new metric reflecting feature importance across all experiments. This new metric was denoted as the adjusted rank (%) hereafter in the text. The first factor (R^1^, ranking of the numeric score) was the minimum value of feature importance computed by extracting the minimum values of IncMSE from 10 random runs, which was ranked such that a lower rank would indicate high importance of the feature across multiple runs. The second factor was based on the coefficient of variation (CV) of the feature importance across 10 random runs, where the factor was also ranked (R^2^, ranking of the CV percentage). A high CV indicates that the variability of feature importance score is high. The third factor (R^3^, ranking of the frequency) was the occurrence of the feature during 10 random runs after feature selection using a correlation filter. The third factor was ranked so that the lowest rank indicates consistent selection of the feature (for example, rank 1 would indicate the presence of the feature in all 10 random runs). In summary, a lower rank of the first factor would indicate a higher feature importance, a lower rank of the second factor would indicate a lower CV of feature importance across multiple random runs, and a lower rank of the third factor would indicate a higher frequency of feature occurrence used to train the models. The ranks were converted to adjusted rank by dividing the sum of the ranks (all factors) for each feature by the sum of maximum ranks within each category (all factors) and subtracting this ratio from one (Eq. 1).


(1) $$R_{F}^{A}=1-\;\frac{\left(R_{F}^{1}+R_{F}^{2}+R_{F}^{3}\right)}{\left(maxR_{F}^{1}+maxR_{F}^{2}+maxR_{F}^{3}\right)}$$

where, $R_{F}^{A}$ is the adjusted rank for each feature; $R_{F}^{1},\;R_{F}^{2},\text{ and }R_{F}^{3}$ are the ranks of each factors; and *max* refers to maximum rank within each category. This equation holds only when the specific feature was selected more than once within multiple runs. The adjusted rank was normalized (*norm_R_F^A^*) and presented as a percent (Eq. 2).


(2) $$norm_{R}F^{A}\left(\%\right)=\;\frac{R_{F}^{A}}{\left(max\;R_{F}^{A}\right)}\times \;100$$

Feature comparison was performed by observing the normalized adjusted rank data.

## 4 Results

### 4.1 Spectral features from remote sensing data and seed yield

**Figure 4:**
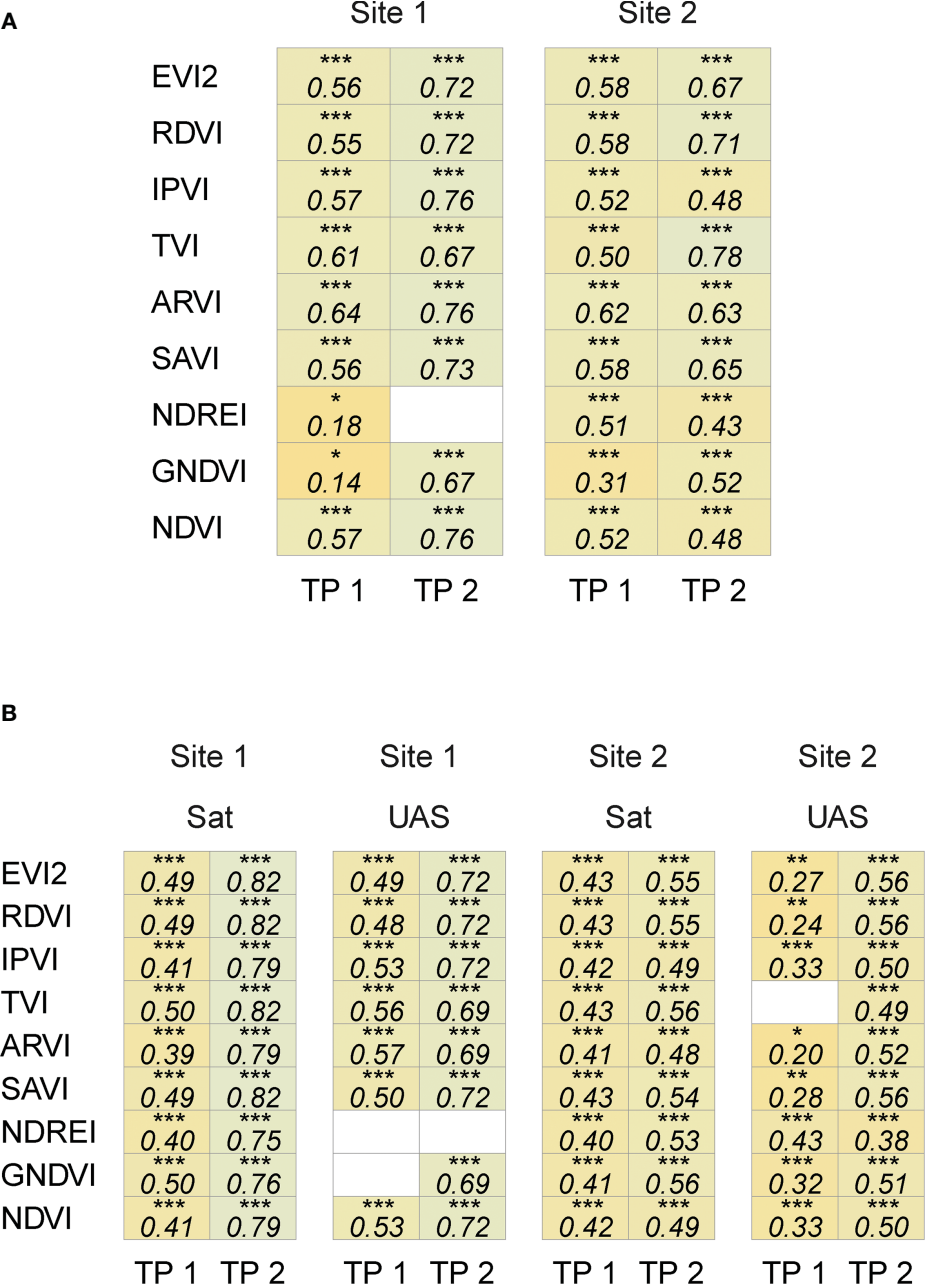
Correlation coefficient between spectral features extracted from satellite and UAS imagery **(A)**, and with seed yield **(B)**. White cells indicate non-significant correlation (p ≥ 0.05), *(0.01< p ≤ 0.05), **(0.001< p ≤ 0.01), and ***(p ≤ 0.001).

The satellite-based features were comparable to UAS-based features in both trials, where the vegetation indices from both sources were significantly and positively correlated (Pearson correlation coefficient, 0.14 ≤ |*r*| ≤ 0.78, p< 0.05) (**Figure 4A**). This relationship (Pearson correlation coefficient = 0.50 ≤ *r* ≤ 0.78 and p< 0.0001) was consistent across time points and sites for eight VIs (except NDREI for Site 1).

The seed yield varied between the two sites as a function of the environment, location, and the evaluated entries. The yield recorded at Site 2 in 2020 (4514 ± 783 kg/ha, n = 131) was higher than at Site 1 in 2019 (1938 ± 489 kg/ha, n = 203). It was encouraging to note that the correlation between the extracted spectral features from satellite and UAS imagery and seed yield data showed similar patterns (**Figure 4B**). Satellite- and UAS-based VIs with the combination of red and NIR bands (e.g., EVI2, RDVI, IPVI, ARVI, SAVI, and NDVI) were significantly and positively correlated with yield data (satellite: 0.38 ≤ *r* ≤ 0.80, UAS: 0.20 ≤ *r* ≤ 0.72, and p< 0.05). Correlation between other spectral features with yield data varied depending on time point (~ crop growth stage) and location. On the other hand, the correlation analysis between texture features extracted from both sensing platforms and seed yield data showed a weak to no correlation (**Supplementary Materials Figure 3**). The relation between these features and seed yield were further assessed in training random forest models to predict the final seed yield in the following sections.

### 4.2 Yield estimation using remote sensing data (Level 1)

#### 4.2.1 Yield estimation using multi-modal data at separate time points

**Figure 5:**
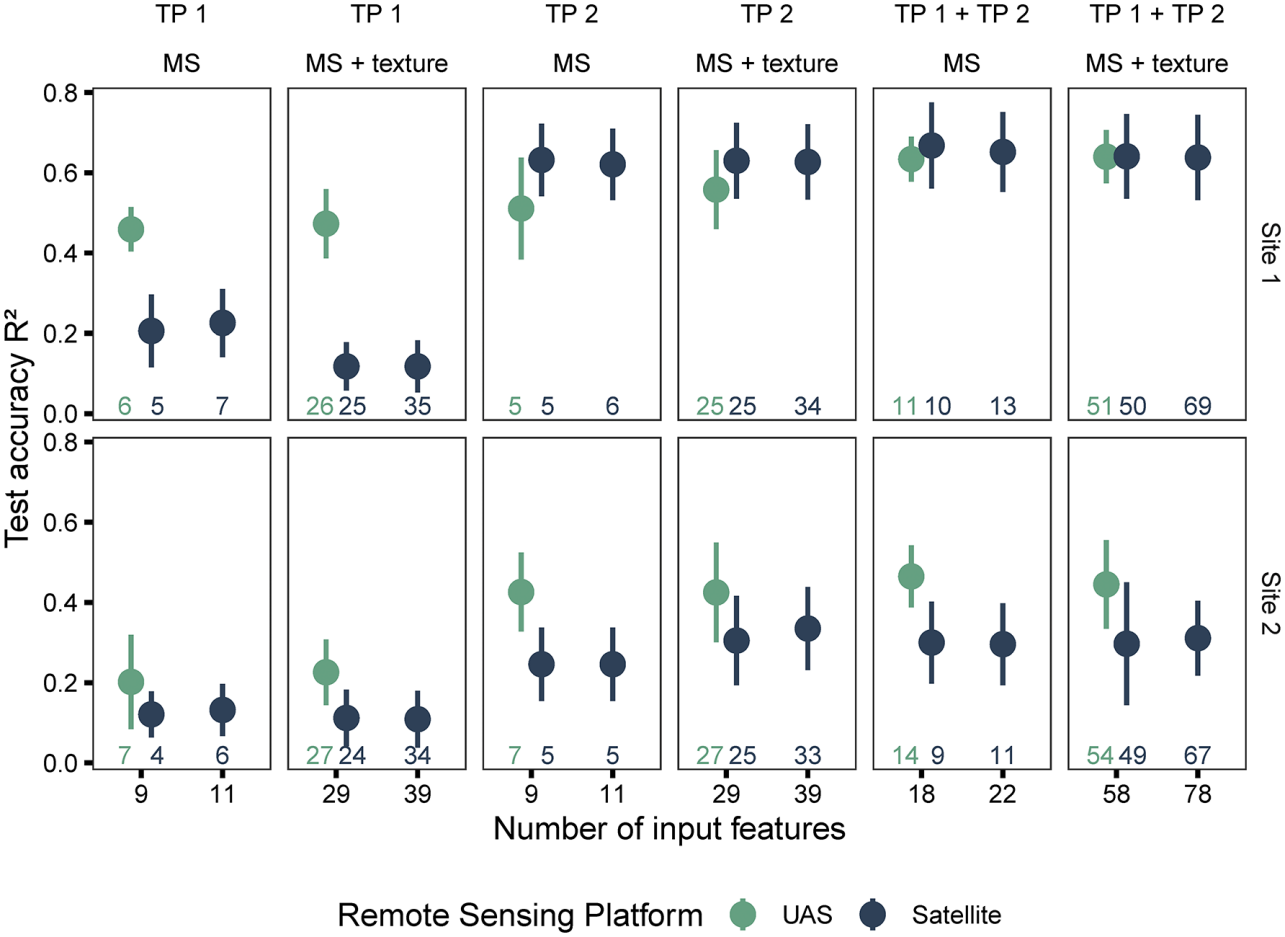
Model performance based on data source (satellite and UAS), type of features used in model (spectral and spectral + texture), and time points (individual and combined time points). The number of input features represented as labels of the x-axis indicates the number of features before the correlation filter. The colored numbers above the x-axis represent the total number of features retained after the correlation filter, which were used to build the random forest models.

During the analysis of individual time points, the satellite- and UAS-based features were evaluated based on the performance of random forest models, trained with these features, to predict harvested seed yield (**Figure 5**; training results and spatial distribution of yield differences in **Supplementary Materials Table 7** and **Figure 4**, respectively). On average, UAS data-based models performed better (higher R^2^, lower RMSE) compared to satellite data-based models (R^2^ = 0.36 ± 0.23; RMSE=523 ± 197 kg/ha for satellite, R^2^ = 0.46 ± 0.16; RMSE=486 ± 173 kg/ha for UAS). However, the difference between the results from the two sensing approaches (two image scales) varied with the crop growth stage during data acquisition (individual time point or combined time points), the breeding trial and its yield variability (Site 1 and Site 2), and the type of features (spectral or spectral + texture) used as input in the models.

Models trained on features extracted from imagery acquired at TP 1 (crop growth stage around flowering at Site 1 and early vegetative growth at Site 2) had weaker performance than TP 2 (pod development). For instance, models developed with features extracted from UAS imagery at TP 2 showed better performance compared to those extracted at TP 1 (R^2^ = 0.25 ± 0.15; RMSE=570 ± 180 kg/ha for TP 1, R^2^ = 0.45 ± 0.17; RMSE=489 ± 189 kg/ha for TP 2). At this growth stage at Site 1 (TP 2), satellite data-based models showed an improved performance compared to UAS data-based models (R^2^ = 0.63 ± 0.09; RMSE=295 ± 37 kg/ha for satellite, R^2^ = 0.53 ± 0.11; RMSE=335 ± 58 kg/ha for UAS). However, at Site 2, satellite data-based models did not outperform UAS data-based models.

Satellite and UAS data-based models performed similarly even when texture features or the two unique bands, yellow (~ 585 – 625 nm) and NIR2 (~ 860 – 1040 nm), were added to the common nine VIs. In both cases (satellite and UAS), the model performances did not improve significantly, although small increases or decreases were occasionally observed (**Figure 5** and **Supplementary Materials Table 7**). Overall, for satellite data-based models, adding texture information reduced the accuracy of models, particularly for the dataset at TP 1. This could be due to the spectral mixing and additional noise. The sample size with respect to the increasing feature space could also contribute to these results.

When the time points were combined, the accuracy of random forest models in predicting seed yield increased in most cases at both sites and for both types of datasets (satellite and UAS). At Site 1, the accuracy of satellite data-based models was slightly better than UAS data-based models (R^2^ = 0.65 ± 0.10; RMSE=284 ± 45 kg/ha for satellite, R^2^ = 0.64 ± 0.06; RMSE=296 ± 55 kg/ha for UAS) when using the combined dataset (TP 1 + TP 2). In fact, combining features from the two time points extracted from satellite or UAS imagery improved the model performance regardless of the image scale (**Figure 5** and **Supplementary Materials Table 7**).

In summary, the satellite data-based models performed poorly compared to UAS-based models, especially at early pea growth stages (prior to canopy closure). The gap in performance was reduced at the later TP 2 and was further improved with combining the data from two time points (especially for Site 1, which could be associated with the crop growth stage). Moreover, the texture information did not improve the performance of the model. Since texture data did not improve the model performance, only spectral data was used for evaluating image and feature fusion henceforth.

#### 4.2.2 Yield estimation using fused features datasets

**Table 2** summarizes the performance of the random forest models resulting from data fusion (three scenarios) of spectral features extracted from satellite and UAS imagery (spatial distribution of yield differences in **Supplementary Materials Figure 5**). The overall trend depicts that combining information from the two sensing approaches improved the prediction accuracy compared to individual time points and/or individual sensors. Particularly, at Site 1, combining features extracted from UAS_TP1_ and Satellite_TP2_ improved the performance of random forest models by a mean increase of R^2^ ranging between 3–50% (decrease in RMSE by 1–26%) compared to the single sensor approach. However, for Site 2, the performance of the random forest models did not improve with integrated features from UAS_TP1_ and Satellite_TP2_ imagery. When compared with the model performance of UAS_TP1+TP2_ dataset, combining the features extracted from the scenario of UAS_TP1_ and Satellite_TP2_ decreased the prediction accuracy (R^2^ = 0.47 ± 0.08; RMSE=587 ± 57 kg/ha for UAS_TP1+TP2_, R^2^ = 0.41 ± 0.14; RMSE=621 ± 75 kg/ha for UAS_TP1_ + Satellite_TP2_). Nevertheless, when all the features extracted from both sources (satellite and UAS) and both time points were integrated, the prediction accuracy surpassed the best result acquired from the individual sensor (UAS_TP1+TP2_) with an increase in R^2^ and a decrease in RMSE. This increase in performance could be attributed to the addition of features extracted from UAS_TP2_.

**Table 2:** Performance evaluation of models using multi-scale/sensor input features.

Site	Scenario	Input/Selected features	Train		Test	
			R^2^	RMSE (kg/ha)	R^2^	RMSE (kg/ha)
1	UAS_TP1_ + Satellite_TP2_	**18/11**	**0.69 ± 0.03**	**284 ± 19**	**0.69 ± 0.09**	**272 ± 53**
	Satellite_TP1_ + UAS_TP2_	18/10	0.57 ± 0.04	331 ± 21	0.58 ± 0.11	316 ± 62
	UAS_TP1+TP2_ + Satellite_TP1+TP2_	**36/21**	**0.72 ± 0.02**	**272 ± 13**	**0.72 ± 0.08**	**259 ± 53**
2	UAS_TP1_ + Satellite_TP2_	18/12	0.35 ± 0.07	661 ± 44	0.41 ± 0.14	621 ± 75
	Satellite_TP1_ + UAS_TP2_	18/11	0.32 ± 0.04	673 ± 24	0.44 ± 0.07	603 ± 55
	UAS_TP1+TP2_ + Satellite_TP1+TP2_	**36/23**	**0.37 ± 0.05**	**638 ± 33**	**0.50 ± 0.11**	**575 ± 70**

The values reported are mean±standard deviation. Bold values indicate the best-performing models.

### 4.3 Evaluation of multi-scale image fusion (Level 2)

#### 4.3.1 Qualitative and quantitative spectral evaluation of pan-sharpened imagery

**Figure 6:**
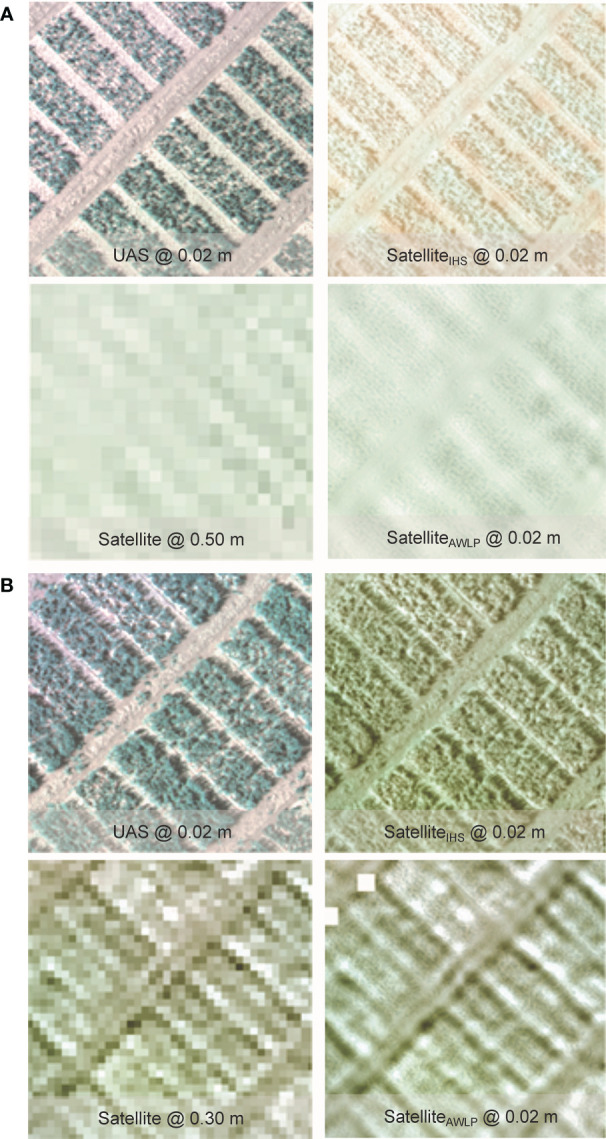
Visual comparison of RGB from the original satellite and UAS imagery, and pan-sharpened satellite imagery generated using IHS and AWLP approaches from Site 1 at TP 1 **(A)** and TP 2 **(B)**.

**Figure 7:**
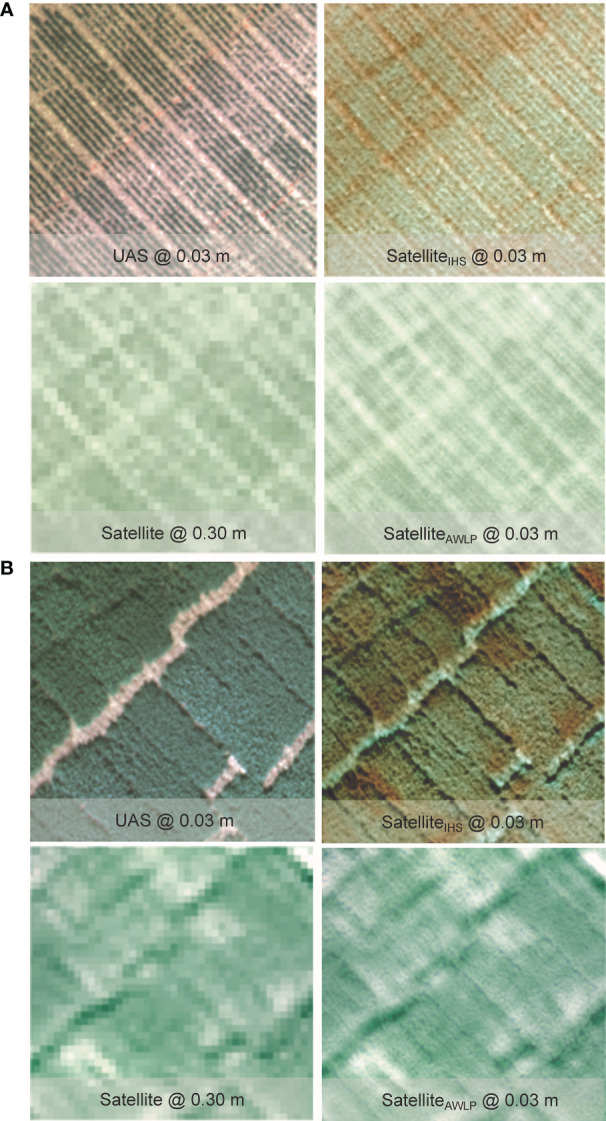
Visual comparison of RGB from the original satellite and UAS imagery, and pan-sharpened satellite imagery generated using intensity-hue-saturation (IHS) and additive wavelet luminance proportional (AWLP) approaches from Site 2 at TP 1 **(A)** and TP 2 **(B)**.

Qualitative evaluation of the pan-sharpening methods was based on visual inspection of the resulting images. **Figures 6** and **7** illustrate image fusion results using UAS as a panchromatic band at their original resolution for each time point at Site 1 and Site 2, respectively (correlation with yield presented in **Supplementary Materials Figure 6**).

Spectral distortion (based on color) can be noted in images obtained using the IHS technique, while the AWLP technique preserved the spectral quality of the satellite imagery in most cases. On the other hand, the spatial quality and resolution of the IHS technique were visually better than the AWLP technique. Overall, we notice that the IHS technique generated images that were very similar to the panchromatic bands (UAS imagery).

**Table 3** summarizes the results of the evaluation metrics of the pan-sharpened imagery. The AWLP method showed comparable image evaluation metrics (CC, SSIM, PSNR, and ERGAS) to the IHS method. The major difference was observed in SAM values, where images pan-sharpened with the AWLP approach had 1.33 times lower SAM values compared to the images pan-sharpened with the IHS approach (averaged over all case studies). This indicates better spectral quality comparison between the pan-sharpened image and reference image (original satellite imagery).

**Table 3:** Image comparison metrics correlation coefficient (CC), the structural similarity index measure (SSIM), the peak signal to noise ratio (PSNR), the erreur relative globale adimensionnelle de synthese (ERGAS), and the spectral angle mapper (SAM) comparing different pan-sharpened imagery (GSD = 0.02-0.03 cm/pixel) generated using intensity-hue-saturation (IHS) and additive wavelet luminance proportional (AWLP) approaches with original satellite imagery.

Site	Original imagery	Technique	CC	SSIM	PSNR	ERGAS	SAM
**1**	UAS_TP1_ + Satellite_TP1_	IHS	0.82	0.77	23.47	2.75	0.87
		AWLP	0.81	0.77	23.21	2.83	0.21
	UAS_TP2_ + Satellite_TP2_	IHS	0.87	0.81	28.27	3.84	1.96
		AWLP	0.88	0.85	28.43	3.72	0.88
**2**	UAS_TP1_ + Satellite_TP1_	IHS	0.84	0.84	25.46	6.92	1.80
		AWLP	0.84	0.85	25.57	6.81	0.43
	UAS_TP2_ + Satellite_TP2_	IHS	0.83	0.77	25.53	7.20	2.90
		AWLP	0.89	0.85	26.72	5.94	0.70

#### 4.3.2 Yield estimation using satellite pan-sharpened imagery

**Figure 8:**
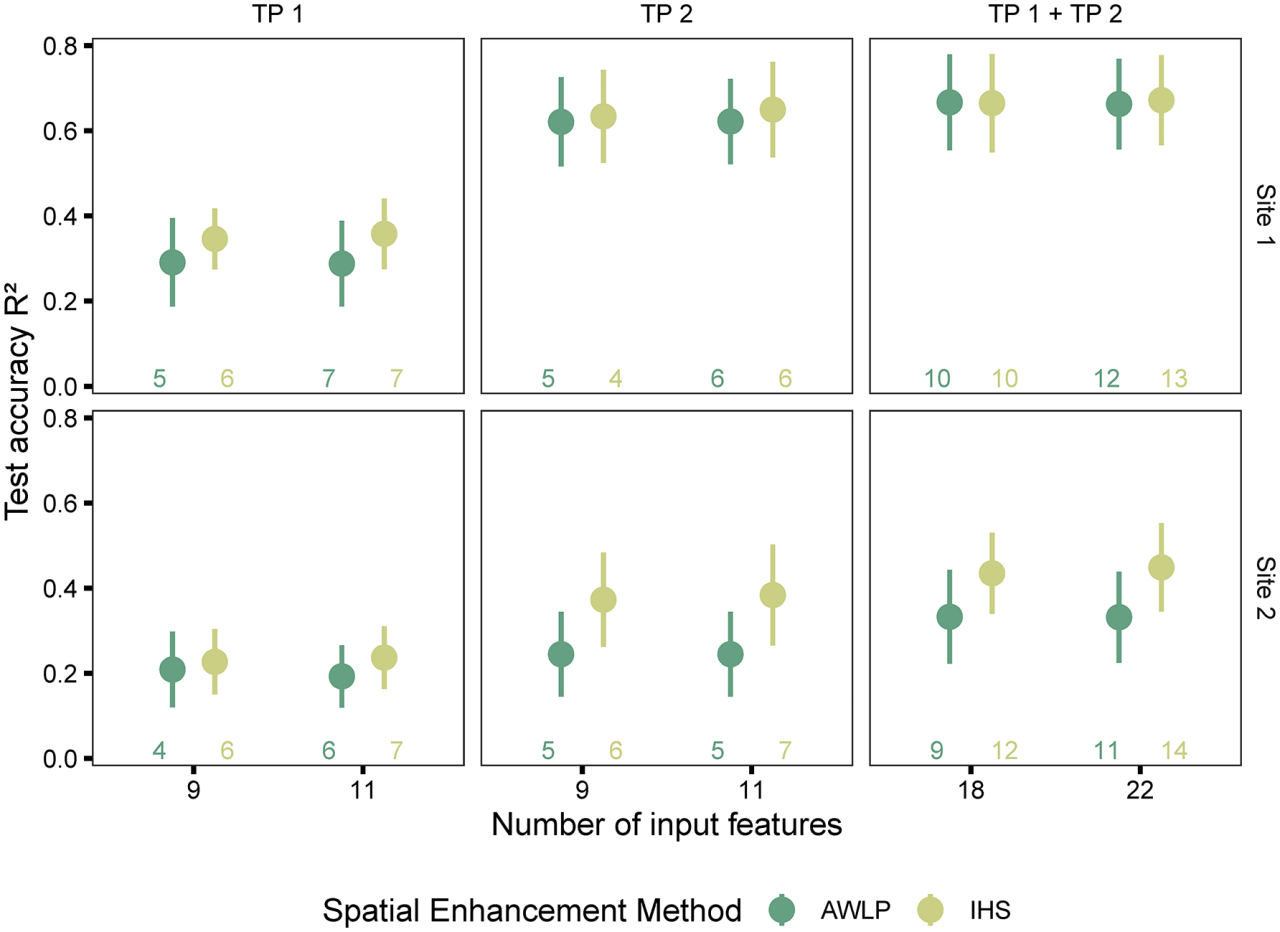
Model performance based on spectral data (pan-sharpened imagery developed using integration of satellite and UAS imagery using AWLP and IHS approaches), and time points (individual and combined time points). The number of input features represented as labels of the x-axis indicates the number of features before the correlation filter. The colored numbers above the x-axis represent the total number of features retained after the correlation filter, which were used to build the random forest models.

In terms of image evaluation metrics, especially SAM, the AWLP approach was better than the IHS approach. However, models trained with features extracted from satellite imagery pan-sharpened with the IHS approach were more accurate in predicting seed yield than models trained with features extracted from satellite imagery pan-sharpened with the AWLP approach (**Figure 8** and **Supplementary Materials Table 8**).

The potential reasons for this observation could include the following: (i) image spatial quality was critical in addition to spectral quality for yield prediction (IHS approach displayed higher spatial quality than AWLP approach), (ii) the image evaluation metrics do not necessarily indicate statistical quantitative assessment (Yokoya et al., 2017, specified in reference to component substitution approach such as IHS transformation), which in this case is yield prediction, (iii) the image evaluation metrics were mainly developed for satellite to satellite image comparisons, and (iv) it was observed that the AWLP approach produced small anomalies during image pan-sharpening that may have affected the results. It should be noted that at Site 1 and with TP 2 and TP 1 + TP 2 datasets, the performance of models built with the features extracted from pan-sharpened imagery with the two approaches did not differ.

When the models were trained with satellite-based features extracted at original resolutions, model performance with features extracted from Satellite*_IHS_* increased, particularly at TP 1. For example, the mean increase in R^2^ ranged between 67% at Site 1 (R^2^ = 0.21 ± 0.09; RMSE=442 ± 62 kg/ha for Satellite_TP1_ at GSD = 0.50 m/pixel, R^2^ = 0.35 ± 0.07; RMSE=397 ± 49 kg/ha for Satellite_TP1_ at GSD = 0.02 m/pixel) to 92% at Site 2 (R^2^ = 0.12 ± 0.06; RMSE=747 ± 47 kg/ha for Satellite_TP1_ at GSD = 0.30 m/pixel, R^2^ = 0.23 ± 0.08; RMSE=702 ± 43 kg/ha for Satellite_TP1_ at GSD = 0.03 m/pixel). Our results show that although the models with pan-sharpened imagery had weaker performance than those obtained from UAS-based models at original resolution, the performance of satellite-derived features, in general, improved after pan-sharpening (spatial enhancement).

### 4.4 Comparison of high definition and pan-sharpened imagery (Level 3)

**Figure 9:**
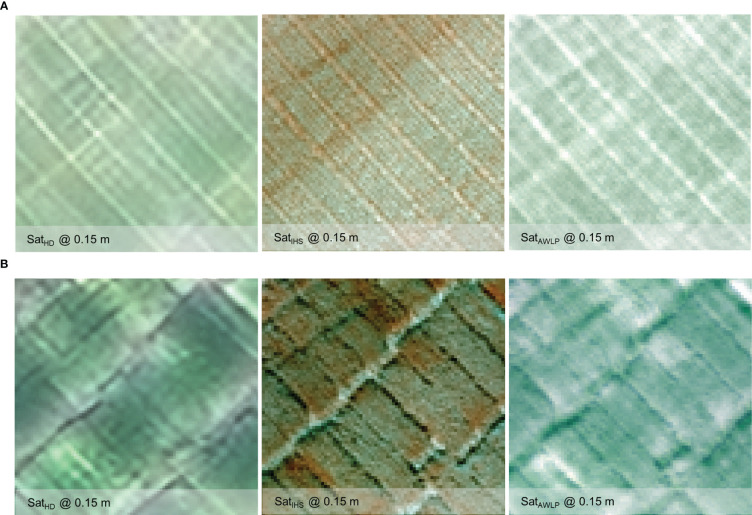
Visual comparison of the high definition (HD) and pan-sharpened satellite imagery generated using intensity-hue-saturation (IHS) and additive wavelet luminance proportional (AWLP) approaches from Site 2 at TP 1 **(A)** and TP 2 **(B)**.

The pan-sharpened imagery was developed by fusing the satellite and UAS images to a resolution (GSD = 15 cm) similar to HD imagery (correlation with yield presented in **Supplementary Materials Figure 7**) for Site 2 datasets. **Figure 9** and **Supplementary Materials Table 9** present the visual representation and spectral and spatial quality metrics variation upon image fusion, respectively. On average, all spectral metrics were comparable between IHS and AWLP approaches, although IHS or AWLP approach may be better in some cases depending on the time points. Nevertheless, in terms of SAM values, the AWLP approach resulted in better results than the IHS approach, similar to the previous section (**Table 3**).

**Figure 10:**
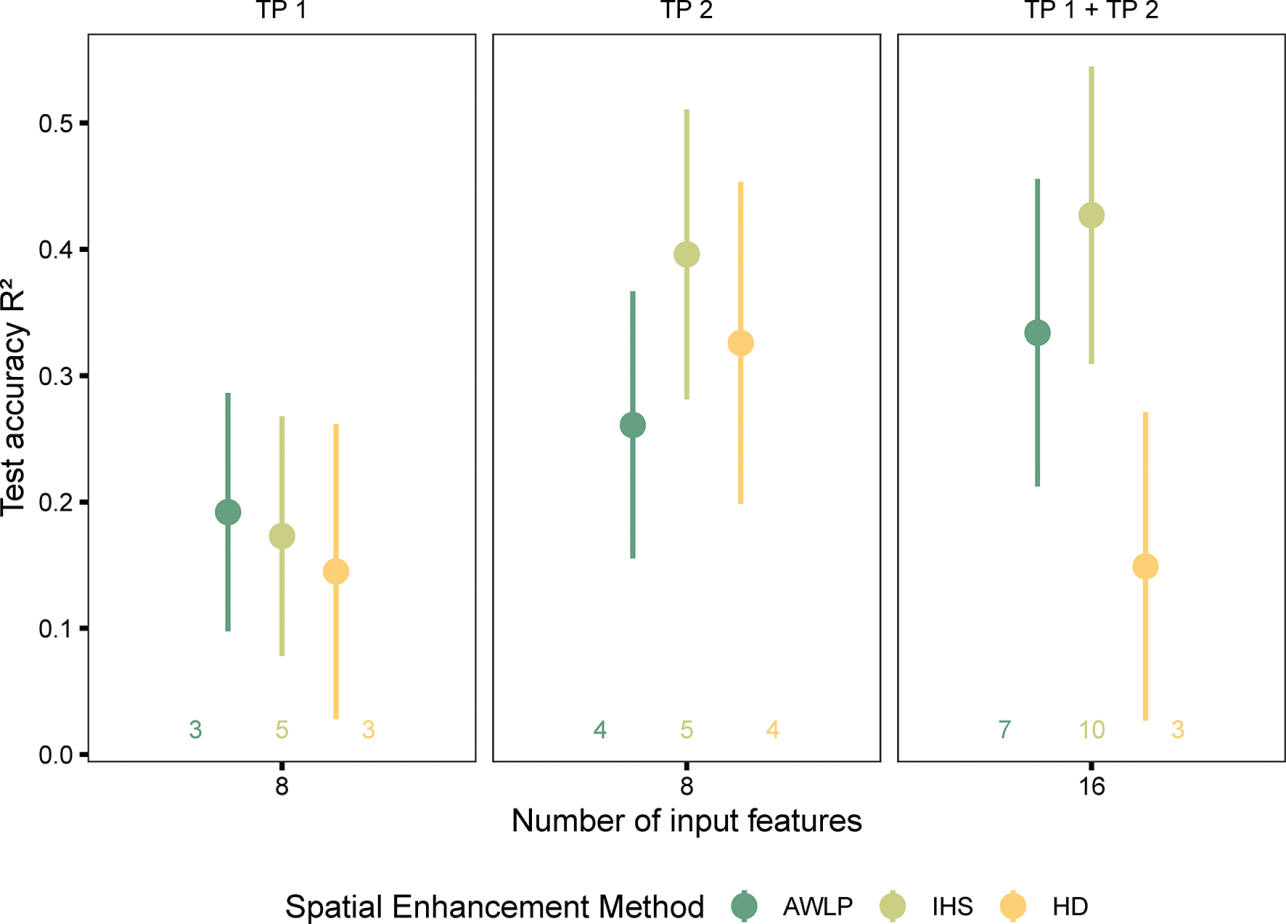
Model performance based on spectral data (pan-sharpened imagery developed using integration of satellite and UAS imagery using AWLP and IHS approaches with GSD = 0.15 m), and time points (individual and combined time points). The number of input features represented as labels of the x-axis indicates the number of features before the correlation filter. The colored numbers above the x-axis represent the total number of features retained after the correlation filter, which were used to build the random forest models.

Comparing image evaluation metrics (with original satellite imagery as reference image) of HD images with pan-sharpened images developed using IHS and AWLP approaches, the CC, SSIM, PSNR, and ERGAS values were better, while SAM values were higher. The yield prediction accuracy (**Figure 10**) varied based on the image fusion approach. The random forest model developed with features extracted from Satellite*_IHS_* showed the highest mean accuracy (especially TP 2 and TP 1 + TP 2) in comparison to the model developed using features extracted from Satellite*_AWLP_* and Satellite*_HD_* images.

As previously discussed, all models evaluated with features extracted from TP 1 imagery performed poorly and are probably associated with the early growth stage. For the combined time points dataset, the model developed with features extracted from the Satellite*_HD_* image performed poorly (**Supplementary Materials Table 10**). The models trained with features extracted from the Satellite*_IHS_* approach gave a comparable performance to those trained with UAS-features (original resolution retrained using the same number of features); with an average increase in R^2^ of only 3% for UAS data-based models. These models outperformed the satellite-based features (original resolution retrained with the same number of features) by 57%.

### 4.5 Feature importance

**Figure 11:**
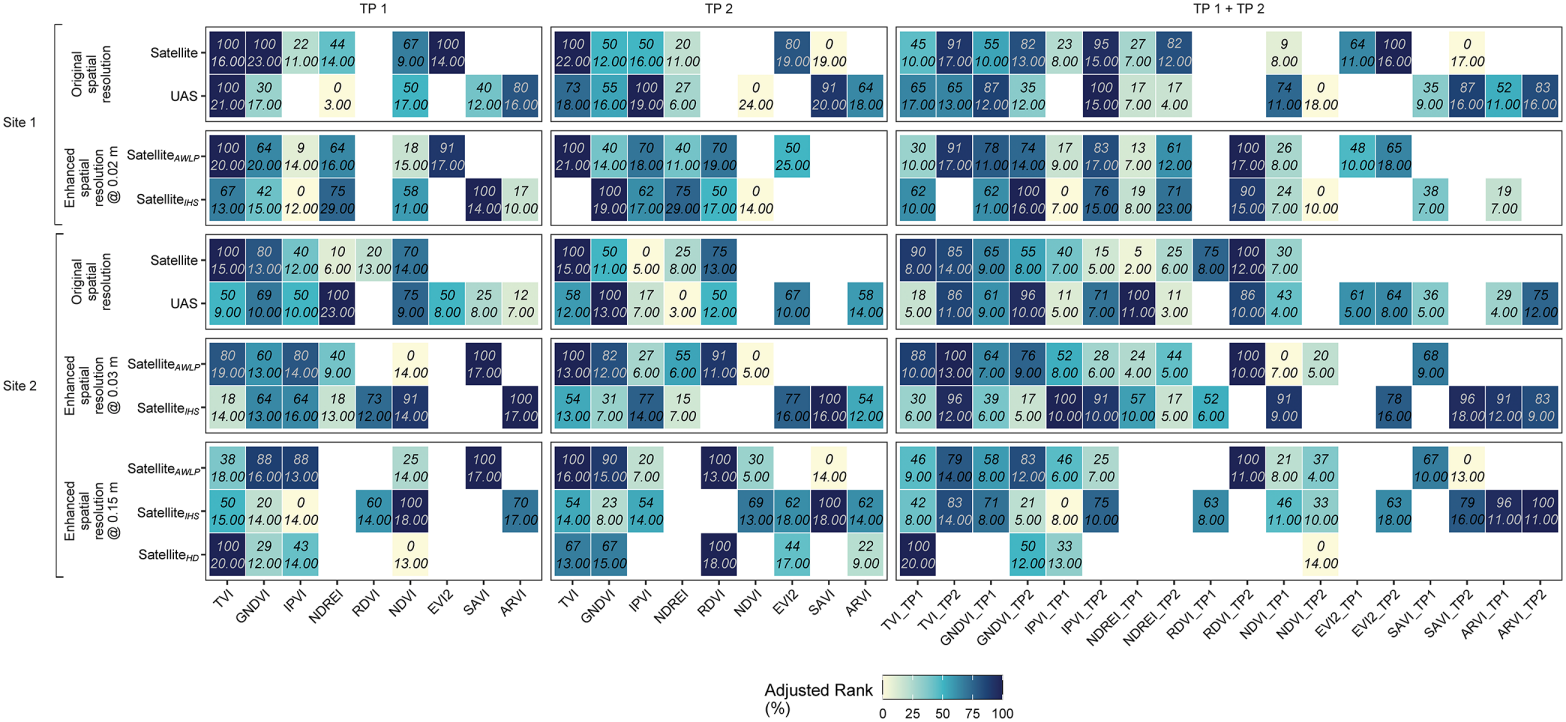
Adjusted rank (%) extracted to evaluate the feature importance from random forest models developed using different datasets. The comparison was made with respect to original (satellite and UAS) and pan-sharpened imagery across multiple time points and trials. In each square, there are two numbers, the top one indicates adjusted rank (%) and bottom one in, *italics*, is indicative of mean importance (%IncMSE).

The evaluation of feature importance based on the adjusted rank takes into account different sources of variability that might influence the assessment instead of relying solely on the feature importance scores (permutation feature importance). These ranks (presented as percentages) reflected feature importance scores, consistency across 10 random runs, and the correlation between features (which was translated as the frequency of occurrence). Therefore, in this section, we focus on reporting the variation of adjusted rank as a metric to capture the stability of these features (**Figure 11**).

Both satellite- and UAS-based models indicate that VIs with NIR and G spectral bands (e.g., GNDVI and TVI; **Supplementary Materials Figure 8**) are the most relevant and consistent features in this study. We found that VIs that are computed with the NIR and R spectral bands are more likely to be collinear (highly correlated with each other) in at least one of the random runs and, as a result, were eliminated before the random forest model training step. With a correlation coefficient threshold of 0.99, SAVI, ARVI, NDVI, RDVI, and EVI2 were found to be highly correlated with each other. When combining features extracted from imagery acquired at both time points, features from TP 2 were selected more frequently during model training, which indicates the importance of early pod development stages for capturing differences in crop performance between entries (Zhang et al., 2021).

## 5 Discussion

UAS-based phenomics is an accurate and efficient tool for providing quality features that supplement traditional field phenotyping. This study demonstrates that high-resolution satellite imagery with a spatial resolution of 0.30 m/pixel can provide quality features as good as UAS-based imagery when evaluating the harvested seed yield of field pea genotypes in small (~9 m^2^) breeding plots at later growth stage. The vegetation indices extracted from satellite imagery can be associated with seed yield at the breeding plot level. These results are in agreement with other studies (Tattaris et al., 2016; Sankaran et al., 2021). However, at earlier growth stages, there was no association between canopy reflectance extracted using satellite imagery and the harvested seed yield, which could be explained by the spatial resolution (pixel size) and the problem of spectral mixing. During this stage, ~50 DAS at Site 2, the pea plants are still in vegetative stage. Spectral mixing with soil adds noise and may reduce the quality of vegetation indices extracted from satellite imagery. Dalla Marta et al. (2015) found similar results when evaluating satellite-based features to estimate the nitrogen concentration of durum wheat at an early stage. In our study, the accuracy of predicting harvested seed yield can be related to the data acquisition time and the crop’s growth stage for breeding plots of 9 m^2^ (25 to 50 pixels per plot) compared to >1000 pixels per plot, as is the case for the UAS imagery. However, another factor that might have played a role in the low model accuracies at Site 2 is the data size. We hypothesized that the features extracted from images are proxies of the plant traits, which follow a non-linear trend with field pea seed yield. The choice of random forest models stems from the fact that this model was extensively used for seed yield prediction in remote sensing applications. The R^2^ during prediction at Site 1 was up to 0.60, with a total number of observations or plots of 203 (data from ~164 plots used to train the models). The lower accuracy at Site 2 can be attributed to small data size (data from ~103 plots used to train the models). Mkhabela et al. (2011) found that the R^2^ between NDVI (extracted from satellite MODIS) and field pea grain yield on the Canadian Prairies was ranging between 0.53 and 0.89, depending on the agro-climatic zone.

When we enhanced the spatial resolution of satellite imagery through pan-sharpening techniques, the accuracy of seed yield prediction increased at both sites compared to the results obtained from models trained with satellite based-features at original resolution. However, it is important to note that it was challenging to separate the soil from the canopy, even with enhanced spatial resolution. Thus, the satellite may be a better option after canopy closure than early growth stages. In future work, we can also explore better approaches to segment the pan-sharpened satellite imagery, such as spectral un-mixing with the assistance of UAS imagery, as reported in Alvarez-Vanhard et al. (2020).

The assessment of feature importance revealed that not all features showed consistency across time points and field pea breeding trials. Sankaran et al. (2021) reported similar results with different features selected for maize yield prediction using UAS and satellite imagery. This change in feature selection between time points can be associated with changes in canopy structure and the resulting changes in reflectance properties. For example, at advanced growth stages, some pea lines develop a taller and denser canopy and, as a result, become more susceptible to lodging, creating more shadowed areas or intertwining with adjacent plots. Many studies have reported that the association of VIs with yield depends on the growth stage (Kyratzis et al., 2017; Fu et al., 2020; Adak et al., 2021; Sankaran et al., 2021; Shafiee et al., 2021). This finding on the importance of the growth stage was further demonstrated by our experiment while investigating the feature fusion approach from multi-scale sensing sources. Even if the integrated features were extracted from different spatial resolution datasets, adding satellite-based features at later growth stages provided more information to capture the change of crop growth dynamics compared to single time point and growth stage models. Fu et al. (2020) and Adak et al. (2021) also found that temporal phenotyping using UAS-based features was more accurate in estimating wheat grain yield.

These insights, in turn, may help to scale-up field phenomics applications. Therefore, to enhance field pea breeding with remote sensing-assisted procedures, high-resolution satellite and UAS can be used separately to derive spectral features associated with yield performance at critical growth stages (flowering and pod filling) or integrated (feature fusion) to provide additional temporal features.

## 6 Summary

High-resolution satellite and UAS-based-multispectral features were evaluated to estimate seed yield using a random forest model in different breeding lines from two diverse (advanced yield and variety testing) trials. Satellite and UAS image features and image fusion approaches were explored in this study. The major potential implications from the study can be described as: (i) High-resolution satellite imagery can be used to estimate seed yield at breeding plot level depending on the growth stage (after canopy closure). (ii) Multi-time points data fusion can be explored to capture crop growth patterns with the temporal features. (iii) And pan-sharpening (multi-source image fusion) is another tool to improve satellite spatial resolution, which could help plant breeders to study historical performance with archived satellite imagery and/or explore satellite hyperspectral imagery (hyper-sharpening) for field-based phenomics.

## Data availability statement

The raw data supporting the conclusions of this article will be made available by the authors, without undue reservation.

## Author contributions

Conceptualization: AM and SS; Data collection: AM, RM, and SV; Data processing and analysis: AM; Resources: SS, RM, and SV; Writing- Original Draft Preparation: AM; Writing – Review & Editing: SS, RM, and SV; Visualization: AM; Supervision: SS; Project Administration: SS, RM, and SV; Funding Acquisition: SS, RM, and SV. All authors contributed to the article and approved the submitted version.
