# Supplementary material for: Remote sensing for field pea yield estimation: A study of multi-scale data fusion approaches in phenomics
Source: Front Plant Sci. 2023 Mar 3;14:1111575. doi: 10.3389/fpls.2023.1111575 (PMC10161932; doi:10.3389/fpls.2023.1111575)
Supplement: Supplementary file 1 [file DataSheet_1.docx]

**Supplementary Materials**

**Table 1.** Spectral bands in remote sensing data and data acquisition dates

| **Remote Sensing Platform** | **Spectral bands** | | **Imagery acquisition date** | **Spatial resolution (m)** |
| --- | --- | --- | --- | --- |
| UAS multispectral | Blue | 465 – 485 (20 nm) | 17 June 2019  16 July 2019 02 June 2020  06 July 2020 | 0.02 – 0.03 |
|  | Green | 550 – 570 (20 nm) |  |  |
|  | Red | 663 – 673 (10 nm) |  |  |
|  | Red edge | 712 – 722 (10 nm) |  |  |
|  | NIR | 820 – 860 (40 nm) |  |  |
| Satellite (WorldView 2/3) | Coastal | 400 – 450 (50 nm) | 11 June 2019 19 July 2019 04 June 2020 24 July 2020 | 0.30 – 0.50 |
|  | Blue | 450 – 510 (60 nm) |  |  |
|  | Green | 510 – 580 (70 nm) |  |  |
|  | Yellow | 585 – 625 (40 nm) |  |  |
|  | Red | 630 – 690 (60 nm) |  |  |
|  | Red edge | 705 – 745 (40 nm) |  |  |
|  | NIR | 770 – 895 (125 nm) |  |  |
|  | NIR2 | 860 – 1040 (180 nm) |  |  |

**Table 2.** Image size after cropping to the extent of region of interest (breeding sites)

| **Site** | **UAS** | **Satellite** | **Satellite HD** |
| --- | --- | --- | --- |
| 1 | 3600x3591 | 158x158; 264x263 |  |
| 2 | 2181x2231 | 223x229 | 447x458 |

**Table 3.** Summary of extracted features from the multispectral imagery. WV in the subscript refers to the features only applicable for satellite imagery based on unique bands. Most vegetation indices were derived from L3Harris Geospatial^1^ with two-band enhanced vegetation index (EVI1) from Rocha and Shaver (2009), and texture analysis from Image Processing Toolbox in Matlab^2^.

| **Sensor** | **Features** | **Formula** |
| --- | --- | --- |
| Multispectral bands (MS) | Normalized difference vegetation index | $NDVI= \frac{NIR-R}{NIR+R}$ |
|  | Green normalized difference vegetation index | $GNDVI= \frac{NIR-G}{NIR+G}$ |
|  | Normalized difference red-edge index | $NDREI= \frac{RE-G}{RE+G}$ |
|  | Soil adjusted vegetation index | $SAVI= 1.5 \times\frac{NIR-R}{NIR+R+0.5}$ |
|  | Atmospherically resistant vegetation index | $ARVI= \frac{NIR-[2\times(R+B)]}{NIR+[2\times(R+B)]}$ |
|  | Transformed triangular vegetation index | $TVI= \frac{120\times\left( NIR-G \right)-200\times(R-G)}{2}$ |
|  | Infrared percentage vegetation index | $IPVI= \frac{NIR}{NIR+R}$ |
|  | Renormalized difference vegetation index | $RDVI= \frac{NIR-R}{\sqrt{NIR+R}}$ |
|  | Two-band enhanced vegetation index | $EVI2=2.5\times\frac{NIR-R}{NIR+(2.4\times R)+1}$ |
| MS_WV_ | Normalized difference red-edge index – with yellow band | $NDRE2= \frac{RE-Y}{RE+Y}$ |
|  | Normalized difference vegetation index – with NIR2 band | $NDVI2= \frac{NIR2-R}{NIR2+R}$ |
| MS + MS_WV_ | Grey-level co-occurrence matrix (GLCM) for each spectral band | CO, HO, CR, EN |

^1^https://www.l3harrisgeospatial.com/docs/vegetationindices.html#:~:text=Vegetation%20Indices%20(VIs)%20are%20combinations,accentuate%20a%20particular%20vegetation%20property; ^2^https://www.mathworks.com/help/images/texture-analysis-using-the-gray-level-co-occurrence-matrix-glcm.html. Rocha, A. V., and Shaver, G. R. (2009). Advantages of a two band EVI calculated from solar and photosynthetically active radiation fluxes. *Agr. For. Meteor*. 149 (9), 1560–1563. doi: 10.1016/j.agrformet.2009.03.016.

**Table 4.** Summary results of the performance of random forest models between data splitting schemes. Models were trained using the common spectral features between Satellite-UAS (n_features_ = 9) as input. The values reported are mean±standard deviation.

| **Holdout method** | **Site** | **Remote sensing platform** | **Time points** | **Selected features** | **Train** | | **Test** | |
| --- | --- | --- | --- | --- | --- | --- | --- | --- |
|  |  |  |  |  | **R^2^** | **RMSE**  **(kg/ha)** | **R^2^** | **RMSE (kg/ha)** |
| Holdout entry | 1 | Satellite | TP 1 | 5 | 0.22±0.03 | 452±17 | 0.21±0.09 | 441±62 |
|  |  |  | TP 2 | 5 | 0.64±0.02 | 304±11 | 0.63±0.09 | 295±34 |
|  |  | UAS | TP 1 | 6 | 0.42±0.02 | 384±14 | 0.46±0.06 | 370±55 |
|  |  |  | TP 2 | 5 | 0.51±0.03 | 353±19 | 0.51±0.13 | 341±62 |
|  | 2 | Satellite | TP 1 | 4 | 0.10±0.01 | 780±21 | 0.12±0.06 | 747±47 |
|  |  |  | TP 2 | 5 | 0.21±0.04 | 727±26 | 0.25±0.09 | 712±84 |
|  |  | UAS | TP 1 | 7 | 0.19±0.04 | 722±24 | 0.20±0.11 | 724±90 |
|  |  |  | TP 2 | 7 | 0.29±0.02 | 677±16 | 0.42±0.10 | 610±72 |
|  | **Average** | | | **6** | **0.32±0.18** | **550±184** | **0.35±0.19** | **530±188** |
| Holdout plot | 1 | Satellite | TP 1 | 5 | 0.22±0.03 | 451±10 | 0.24±0.10 | 436±41 |
|  |  |  | TP 2 | 5 | 0.65±0.02 | 301±10 | 0.63±0.08 | 302±32 |
|  |  | UAS | TP 1 | 6 | 0.42±0.03 | 386±11 | 0.47±0.12 | 360±47 |
|  |  |  | TP 2 | 5 | 0.52±0.03 | 349±10 | 0.51±0.12 | 347±40 |
|  | 2 | Satellite | TP 1 | 4 | 0.13±0.04 | 756±24 | 0.09±0.08 | 783±82 |
|  |  |  | TP 2 | 5 | 0.21±0.07 | 719±38 | 0.26±0.15 | 706±107 |
|  |  | UAS | TP 1 | 7 | 0.23±0.03 | 702±21 | 0.20±0.12 | 713±78 |
|  |  |  | TP 2 | 7 | 0.35±0.04 | 646±27 | 0.26±0.14 | 689±96 |
|  | **Average** | | | **6** | **0.34±0.17** | **539±176** | **0.33±0.20** | **542±199** |

**Table 5.** Summary of sample size and seed yield in train and test data used during data splitting schemes. The values reported are mean±standard deviation.

|  | **Site 1** | | | | | | **Site 2** | | | | | |
| --- | --- | --- | --- | --- | --- | --- | --- | --- | --- | --- | --- | --- |
|  | **Train (80%)** | | | **Test (20%)** | | | **Train (80%)** | | | **Test (20%)** | | |
|  | **n_total_** | **n_entry_** | **Yield (kg/ha)** | **n_total_** | **n_entry_** | **Yield (kg/ha)** | **n_total_** | **n_entry_** | **Yield (kg/ha)** | **n_total_** | **n_entry_** | **Yield (kg/ha)** |
| Random holdout plot | | | | | | | | | | | | |
| 1 | 164 | 64 | 1943±500 | 39 | 33 | 1918±495 | 107 | 33 | 4518±781 | 24 | 19 | 4496±811 |
| 2 | 164 | 65 | 1936±504 | 39 | 32 | 1947±478 | 107 | 33 | 4504±771 | 24 | 15 | 4559±851 |
| 3 | 164 | 65 | 1940±492 | 39 | 32 | 1929±531 | 107 | 33 | 4505±797 | 24 | 18 | 4553±736 |
| 4 | 164 | 65 | 1952±493 | 39 | 31 | 1879±521 | 107 | 33 | 4527±796 | 24 | 20 | 4456±740 |
| 5 | 164 | 64 | 1949±497 | 39 | 30 | 1891±506 | 107 | 33 | 4523±777 | 24 | 18 | 4476±828 |
| 6 | 164 | 64 | 1939±506 | 39 | 32 | 1934±471 | 107 | 32 | 4491±783 | 24 | 17 | 4619±793 |
| 7 | 164 | 65 | 1948±498 | 39 | 33 | 1897±504 | 107 | 33 | 4515±774 | 24 | 19 | 4509±841 |
| 8 | 164 | 64 | 1941±508 | 39 | 34 | 1927±462 | 107 | 33 | 4511±774 | 24 | 18 | 4529±841 |
| 9 | 164 | 65 | 1938±507 | 39 | 32 | 1940±466 | 107 | 33 | 4521±778 | 24 | 20 | 4485±823 |
| 10 | 164 | 64 | 1934±498 | 39 | 33 | 1958±503 | 107 | 33 | 4510±813 | 24 | 18 | 4534±652 |
| Random holdout entry | | | | | | | | | | | | |
| 1 | 161 | 52 | 1979±509 | 42 | 13 | 1782±424 | 103 | 26 | 4446±771 | 28 | 7 | 4766±792 |
| 2 | 162 | 52 | 1876±488 | 41 | 13 | 2186±463 | 103 | 26 | 4494±804 | 28 | 7 | 4589±711 |
| 3 | 164 | 52 | 1948±513 | 39 | 13 | 1899±434 | 103 | 26 | 4498±784 | 28 | 7 | 4575±792 |
| 4 | 161 | 52 | 1935±487 | 42 | 13 | 1950±543 | 103 | 26 | 4557±803 | 28 | 7 | 4355±699 |
| 5 | 164 | 52 | 1904±513 | 39 | 13 | 2082±404 | 103 | 26 | 4541±791 | 28 | 7 | 4417±759 |
| 6 | 164 | 52 | 1918±504 | 39 | 13 | 2024±469 | 104 | 26 | 4559±780 | 27 | 7 | 4340±786 |
| 7 | 164 | 52 | 1941±511 | 39 | 13 | 1927±447 | 103 | 26 | 4512±739 | 28 | 7 | 4523±945 |
| 8 | 164 | 52 | 1947±456 | 39 | 13 | 1903±652 | 103 | 26 | 4444±782 | 28 | 7 | 4772±747 |
| 9 | 161 | 52 | 1962±505 | 42 | 13 | 1848±466 | 103 | 26 | 4567±784 | 28 | 7 | 4321±763 |
| 10 | 164 | 52 | 1924±516 | 39 | 13 | 1999±414 | 103 | 26 | 4588±797 | 28 | 7 | 4242±678 |

**Table 6.** Input features used to develop and evaluate random forest models to compare the performance of raw data and datasets derived from multi-scale image and feature fusion approaches.

| Evaluation | Spectral Input Features |
| --- | --- |
| *Spectral features from satellite and UAS data* | |
| Individual sensor raw data (at original resolution satellite and UAS imagery) – each time point^1^ | 9 MS UAS  11 MS + MS_wv_ satellite |
| Individual sensor raw data (at original resolution satellite and UAS imagery) – combined time points | 9 MS x 2 time points = 18 UAS  11 MS + MS_wv_ x 2 time points = 22 satellite |
| Combined sensor data (at original resolution satellite and UAS imagery) – each time point^2^ | 9 MS x 2 sensors = 18 |
| Combined sensor data (at original resolution satellite and UAS imagery) – combined time points | 9 MS x 2 sensors x 2 time points = 36 |
| Pan-sharpened image data (0.02-0.03 m/pixels) – each time point | 9 MS  11 MS + MS_wv_ |
| Pan-sharpened image data (0.02-0.03 m/pixels) – combined time points | 9 MS x 2 time points = 18  11 MS + MS_wv_ x 2 time points = 22 |
| Pan-sharpened image data (0.15 m/pixels) – each time point^3^ | 8 MS |
| Pan-sharpened image data (0.15 m/pixels) – combined time points^3^ | 8 MS x 2 time points = 16 |
| HD image data (0.15 m/pixels) – each time point^3^ | 8 MS |
| HD image data (0.15 m/pixels) – combined time points^3^ | 8 MS x 2 time points = 16 |
| *Integration of spectral and texture features from satellite and UAS data* | |
| Individual sensor raw data (at original resolution satellite imagery) – each time point | 11 MS + 7 spectral bands x 4 texture features = 39 satellite |
| Individual sensor raw data (at original resolution UAS imagery) – each time point | 9 MS + 5 spectral bands x 4 texture features = 29 UAS |
| Individual sensor raw data (at original resolution satellite – combined time points | 39 x 2 time points = 78 satellite |
| Individual sensor raw data (at original resolution UAS imagery) – combined time points | 29 x 2 time points = 58 UAS |

^1^The same dataset was used to assess plot holdout and entry holdout approaches; ^2^For combined sensor data, only common VIs were utilized, for individual time points – the data sources were mixed and matched, for example TP 1 from UAS dataset was integrated with TP 2 from satellite dataset; ^3^Normalized difference red-edge index was not included since HD image only comprised 4 bands without red-edge band. MS refers to the vegetation indices extracted from multispectral band.

**Table 7.** Variation of model performance by sensor, type of input features, time points and sites. Features were extracted from imagery at their original spatial resolution. The values reported are mean±standard deviation.

| **Site** | **Feature Type** | **Remote sensing platform** | **Time points** | **Selected**  **features** | **Train** | | **Test** | |
| --- | --- | --- | --- | --- | --- | --- | --- | --- |
|  |  |  |  |  | **R^2^** | **RMSE**  **(kg/ha)** | **R^2^** | **RMSE (kg/ha)** |
| 1 | MS | Satellite | TP 1 | 5 | 0.22±0.02 | 452±17 | 0.21±0.09 | 442±62 |
|  |  |  | TP 2 | 5 | 0.64±0.02 | 303±12 | 0.63±0.09 | 295±34 |
|  |  |  | TP 1 + TP 2 | 10 | 0.67±0.03 | 297±15 | 0.67±0.11 | 276±46 |
|  |  | UAS | TP 1 | 6 | 0.41±0.02 | 386±13 | 0.46±0.06 | 370±55 |
|  |  |  | TP 2 | 5 | 0.51±0.04 | 352±20 | 0.51±0.13 | 341±62 |
|  |  |  | TP 1 + TP 2 | 11 | 0.63±0.02 | 313±21 | 0.63±0.06 | 298±60 |
|  | MS + texture | Satellite | TP 1 | 25 | 0.16±0.02 | 463±16 | 0.12±0.06 | 463±58 |
|  |  |  | TP 2 | 25 | 0.62±0.02 | 309±12 | 0.63±0.09 | 293±42 |
|  |  |  | TP 1 + TP 2 | 50 | 0.64±0.04 | 304±21 | 0.64±0.11 | 288±45 |
|  |  | UAS | TP 1 | 26 | 0.45±0.03 | 374±16 | 0.47±0.09 | 362±60 |
|  |  |  | TP 2 | 25 | 0.55±0.03 | 341±18 | 0.56±0.10 | 329±57 |
|  |  |  | TP 1 + TP 2 | 51 | 0.61±0.02 | 318±15 | 0.64±0.07 | 293±52 |
|  | MS_WV_ | Satellite | TP 1 | 7 | 0.24±0.02 | 446±18 | 0.23±0.08 | 433±58 |
|  |  |  | TP 2 | 6 | 0.64±0.02 | 305±11 | 0.62±0.09 | 298±34 |
|  |  |  | TP 1 + TP 2 | 13 | 0.65±0.03 | 299±16 | 0.65±0.10 | 283±46 |
|  | MS_WV_ + texture | Satellite | TP 1 | 35 | 0.16±0.02 | 466±13 | 0.12±0.06 | 464±58 |
|  |  |  | TP 2 | 34 | 0.62±0.02 | 309±13 | 0.63±0.09 | 295±43 |
|  |  |  | TP 1 + TP 2 | 69 | 0.63±0.03 | 306±15 | 0.64±0.11 | 290±47 |
| 2 | MS | Satellite | TP 1 | 4 | 0.10±0.02 | 778±17 | 0.12±0.06 | 747±47 |
|  |  |  | TP 2 | 5 | 0.21±0.05 | 725±30 | 0.25±0.09 | 712±84 |
|  |  |  | TP 1 + TP 2 | 9 | 0.22±0.06 | 726±30 | 0.30±0.10 | 679±73 |
|  |  | UAS | TP 1 | 7 | 0.20±0.04 | 724±20 | 0.20±0.12 | 720±93 |
|  |  |  | TP 2 | 7 | 0.29±0.02 | 676±21 | 0.43±0.10 | 609±72 |
|  |  |  | TP 1 + TP 2 | 14 | 0.37±0.06 | 641±39 | 0.47±0.08 | 587±57 |
|  | MS + texture | Satellite | TP 1 | 24 | 0.11±0.03 | 749±22 | 0.11±0.07 | 739±67 |
|  |  |  | TP 2 | 25 | 0.28±0.05 | 679±22 | 0.30±0.11 | 672±62 |
|  |  |  | TP 1 + TP 2 | 49 | 0.28±0.07 | 692±28 | 0.30±0.15 | 672±57 |
|  |  | UAS | TP 1 | 27 | 0.19±0.02 | 717±21 | 0.23±0.08 | 695±59 |
|  |  |  | TP 2 | 27 | 0.31±0.04 | 660±21 | 0.42±0.12 | 618±93 |
|  |  |  | TP 1 + TP 2 | 54 | 0.32±0.03 | 665±30 | 0.44±0.11 | 611±88 |
|  | MS_WV_ | Satellite | TP 1 | 6 | 0.10±0.02 | 779±22 | 0.13±0.06 | 747±48 |
|  |  |  | TP 2 | 5 | 0.22±0.05 | 724±30 | 0.25±0.09 | 712±84 |
|  |  |  | TP 1 + TP 2 | 11 | 0.25±0.06 | 711±27 | 0.30±0.10 | 684±69 |
|  | MS_WV_ + texture | Satellite | TP 1 | 34 | 0.11±0.02 | 753±19 | 0.11±0.07 | 741±67 |
|  |  |  | TP 2 | 33 | 0.31±0.03 | 662±13 | 0.34±0.10 | 652±54 |
|  |  |  | TP 1 + TP 2 | 67 | 0.31±0.07 | 675±31 | 0.31±0.09 | 669±64 |

**Table 8.** Variation of model performance by satellite input features at different time points and sites. Features were extracted from satellite imagery pan-sharpened using IHS and AWLP. The values reported are mean±standard deviation.

| **Site** | **Feature type** | **Pan-sharpening method** | **Time points** | **Selected**  **features** | **Train** | | **Test** | |
| --- | --- | --- | --- | --- | --- | --- | --- | --- |
|  |  |  |  |  | **R^2^** | **RMSE**  **(kg/ha)** | **R^2^** | **RMSE (kg/ha)** |
| 1 | MS | AWLP | TP 1 | 5 | 0.24±0.04 | 451±20 | 0.29±0.10 | 414±59 |
|  |  |  | TP 2 | 5 | 0.64±0.03 | 305±16 | 0.62±0.10 | 296±33 |
|  |  |  | TP 1 + TP 2 | 10 | 0.67±0.03 | 293±13 | 0.67±0.11 | 277±45 |
|  |  | IHS | TP 1 | 6 | 0.36±0.04 | 407±22 | 0.35±0.07 | 397±49 |
|  |  |  | TP 2 | 4 | 0.67±0.03 | 289±12 | 0.63±0.11 | 292±37 |
|  |  |  | TP 1 + TP 2 | 10 | 0.69±0.03 | 286±14 | 0.66±0.12 | 278±47 |
|  | MS_WV_ | AWLP | TP 1 | 7 | 0.27±0.04 | 437±23 | 0.29±0.10 | 412±56 |
|  |  |  | TP 2 | 6 | 0.64±0.03 | 306±15 | 0.62±0.10 | 296±30 |
|  |  |  | TP 1 + TP 2 | 12 | 0.66±0.04 | 299±12 | 0.66±0.11 | 278±45 |
|  |  | IHS | TP 1 | 7 | 0.37±0.05 | 406±22 | 0.36±0.08 | 388±51 |
|  |  |  | TP 2 | 6 | 0.67±0.03 | 292±14 | 0.65±0.11 | 286±44 |
|  |  |  | TP 1 + TP 2 | 13 | 0.69±0.02 | 283±15 | 0.67±0.11 | 274±48 |
| 2 | MS | AWLP | TP 1 | 4 | 0.14±0.06 | 770±42 | 0.21±0.09 | 720±52 |
|  |  |  | TP 2 | 5 | 0.24±0.08 | 714±29 | 0.24±0.10 | 706±90 |
|  |  |  | TP 1 + TP 2 | 9 | 0.25±0.06 | 717±29 | 0.33±0.11 | 664±84 |
|  |  | IHS | TP 1 | 6 | 0.16±0.05 | 757±38 | 0.23±0.08 | 702±43 |
|  |  |  | TP 2 | 6 | 0.35±0.06 | 666±20 | 0.37±0.11 | 648±46 |
|  |  |  | TP 1 + TP 2 | 12 | 0.39±0.06 | 644±32 | 0.44±0.10 | 612±55 |
|  | MS_WV_ | AWLP | TP 1 | 6 | 0.15±0.04 | 771±42 | 0.19±0.07 | 726±55 |
|  |  |  | TP 2 | 5 | 0.25±0.07 | 711±29 | 0.24±0.10 | 706±90 |
|  |  |  | TP 1 + TP 2 | 11 | 0.25±0.07 | 734±57 | 0.33±0.11 | 659±83 |
|  |  | IHS | TP 1 | 7 | 0.18±0.05 | 745±35 | 0.24±0.07 | 694±42 |
|  |  |  | TP 2 | 7 | 0.36±0.06 | 665±34 | 0.38±0.12 | 646±49 |
|  |  |  | TP 1 + TP 2 | 14 | 0.04±0.08 | 634±40 | 0.45±0.10 | 601±56 |

**Table 9.** Image comparison metrics correlation coefficient (CC), the structural similarity index measure (SSIM), the peak signal to noise ratio (PSNR), the erreur relative globale adimensionnelle de synthese (ERGAS), and the spectral angle mapper (SAM) comparing different pan-sharpened imagery (GSD = 15 cm/pixel) generated using intensity-hue-saturation (IHS) and additive wavelet luminance proportional (AWLP) approaches and high definition (HD) imagery with original satellite imagery.

| Original imagery | Techniques | CC | SSIM | PSNR | ERGAS | SAM |
| --- | --- | --- | --- | --- | --- | --- |
| UAS_TP1_ + Satellite_TP1_ | IHS | 0.85 | 0.85 | 25.84 | 32.35 | 1.82 |
|  | AWLP | 0.81 | 0.84 | 24.60 | 37.05 | 0.63 |
| UAS_TP2_ + Satellite_TP2_ | IHS | 0.84 | 0.77 | 25.84 | 34.63 | 3.03 |
|  | AWLP | 0.86 | 0.81 | 25.79 | 32.02 | 1.15 |
| Satellite_TP1_ | HD | 0.92 | 0.89 | 30.15 | 24.40 | 5.48 |
| Satellite_TP2_ | HD | 0.93 | 0.86 | 31.24 | 23.37 | 3.17 |

**Table 10.** Variation of model performance by satellite input features at different time points at Site 2. Features were extracted from satellite HD imagery, satellite imagery pan-sharpened using IHS and AWLP, and UAS and satellite at original resolution (RGB+NIR). The values reported are mean±standard deviation.

| **Method** | **Time points** | **Selected**  **features** | **Train** | | **Test** | |
| --- | --- | --- | --- | --- | --- | --- |
|  |  |  | **R^2^** | **RMSE**  **(kg/ha)** | **R^2^** | **RMSE (kg/ha)** |
| AWLP | TP 1 | 3 | 0.14±0.04 | 791±26 | 0.19±0.09 | 737±77 |
|  | TP 2 | 4 | 0.24±0.06 | 718±52 | 0.26±0.11 | 698±84 |
|  | TP 1 + TP 2 | 7 | 0.30±0.06 | 694±33 | 0.33±0.12 | 660±84 |
| IHS | TP 1 | 5 | 0.15±0.06 | 781±47 | 0.17±0.09 | 737±64 |
|  | TP 2 | 5 | 0.33±0.05 | 662±22 | 0.40±0.11 | 637±51 |
|  | TP 1 + TP 2 | 10 | 0.38±0.06 | 629±21 | 0.43±0.12 | 620±58 |
| HD | TP 1 | 3 | 0.11±0.04 | 785±45 | 0.15±0.12 | 755±64 |
|  | TP 2 | 4 | 0.28±0.08 | 698±36 | 0.33±0.13 | 660±71 |
|  | TP 1 + TP 2 | 3 | 0.15±0.05 | 771±39 | 0.15±0.12 | 754±64 |
| Satellite | TP 1 | 3 | 0.10±0.01 | 790±22 | 0.11±0.05 | 756±43 |
|  | TP 2 | 4 | 0.22±0.04 | 720±28 | 0.23±0.09 | 720±88 |
|  | TP 1 + TP 2 | 7 | 0.24±0.05 | 704±20 | 0.30±0.09 | 674±63 |
| UAS | TP 1 | 6 | 0.14±0.03 | 748±27 | 0.15±0.09 | 735±61 |
|  | TP 2 | 6 | 0.29±0.02 | 674±17 | 0.42±0.10 | 616±69 |
|  | TP 1 + TP 2 | 12 | 0.36±0.07 | 656±45 | 0.46±0.07 | 590±54 |


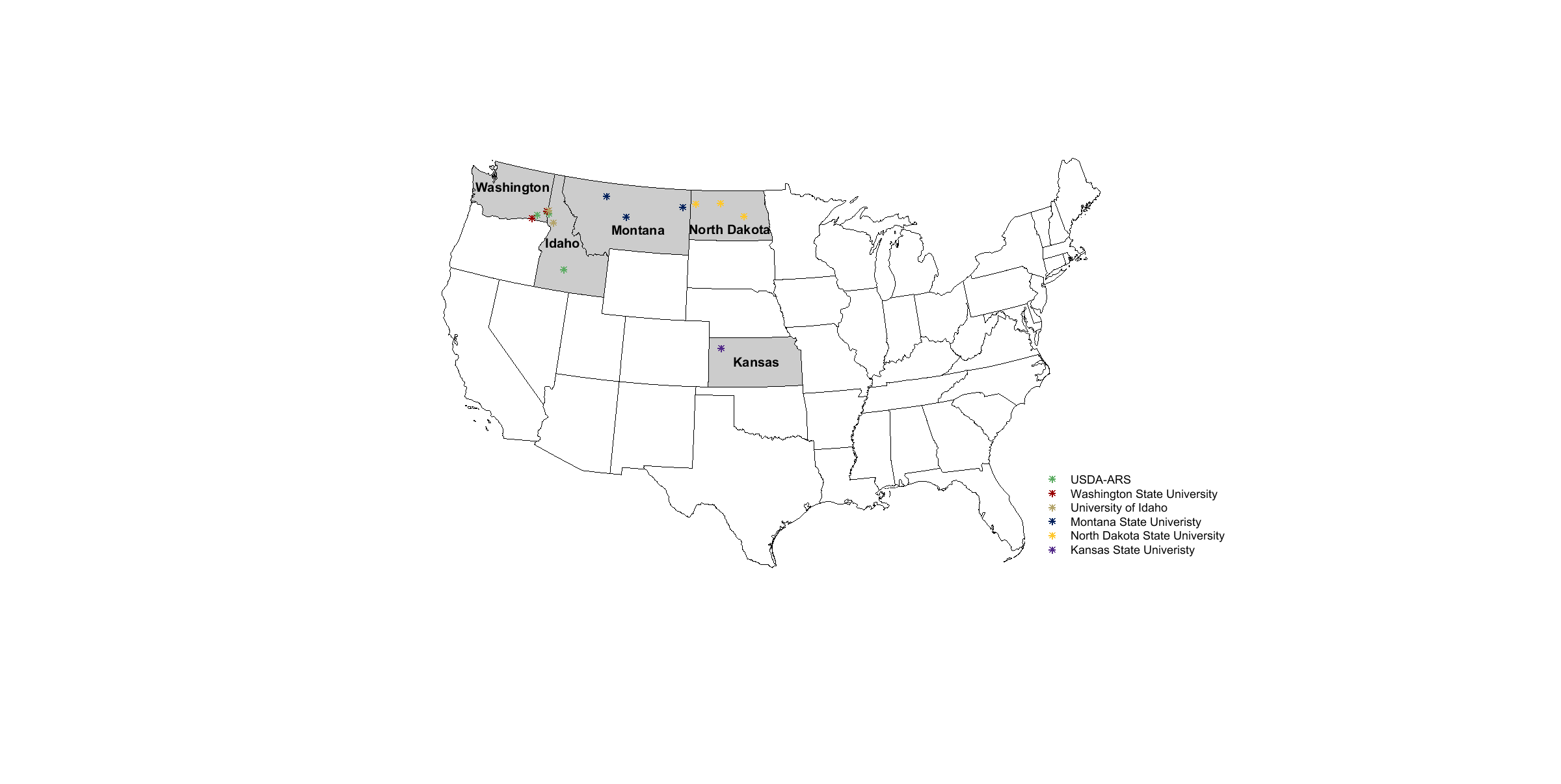


**Fig. 1.** Breeding trial locations of pea breeding program in the Pacific Northwest USA.


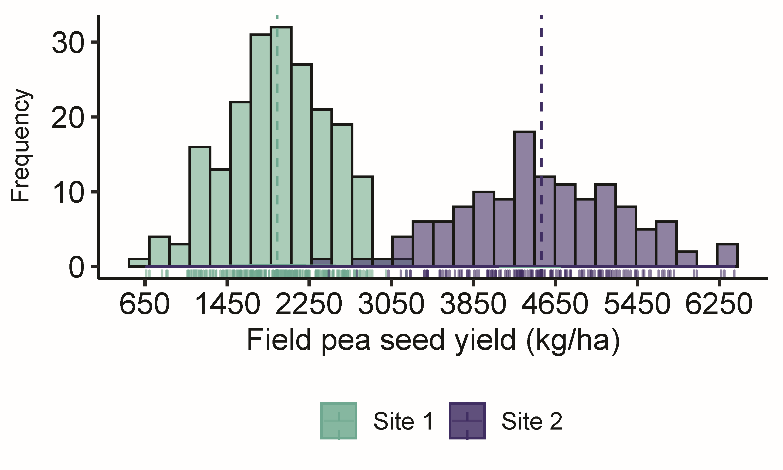


**Fig. 2.** Normal distribution of pea seed yield in advanced yield (Site 1) and variety testing (Site 2) trials.


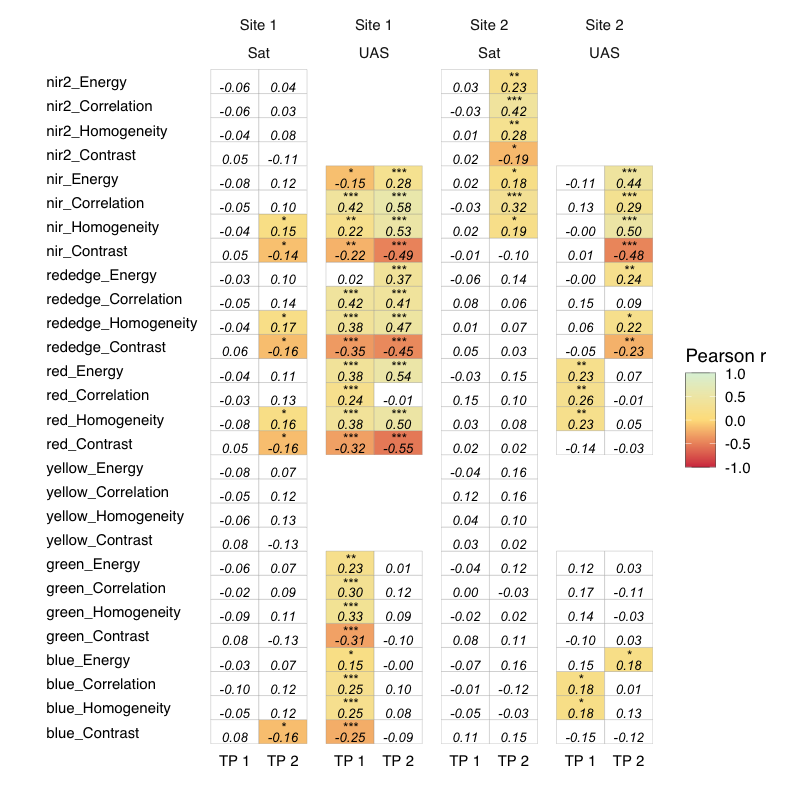


**Fig. 3.** Correlation coefficient between texture features extracted from satellite and UAS imagery with seed yield data at original spatial resolutions. White cells indicate non-significant correlation (p ≥ 0.05), * (0.01 < p ≤ 0.05), ** (0.001 < p ≤ 0.01), and *** (p ≤ 0.001).


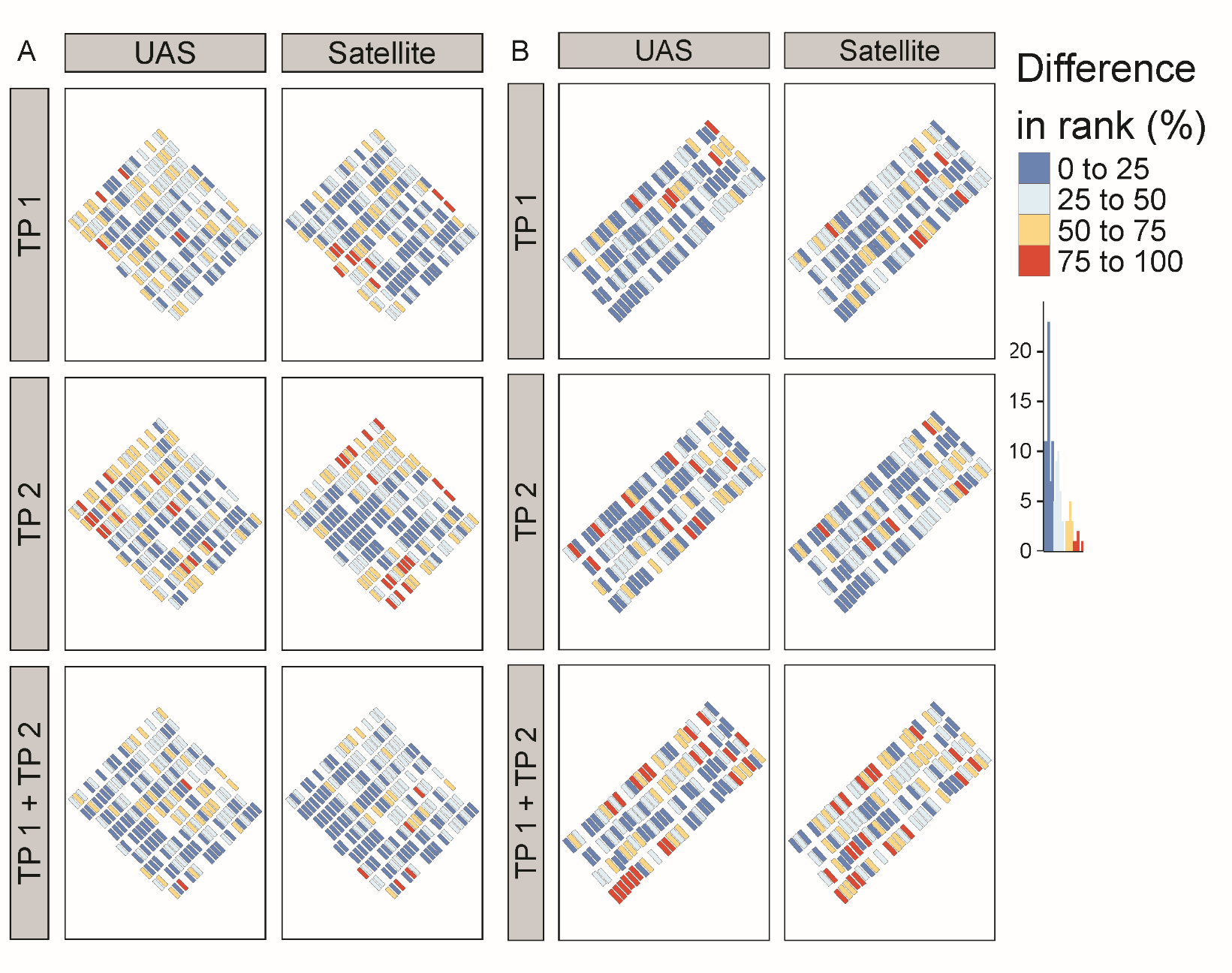


**Fig. 4.** Difference in relative ranking between actual and predicted seed yield using random forest models with UAS and satellite features from Site 1 (A) and Site 2 (B) (overall model performance presented in Fig. 5 in the manuscript, spectral features only).


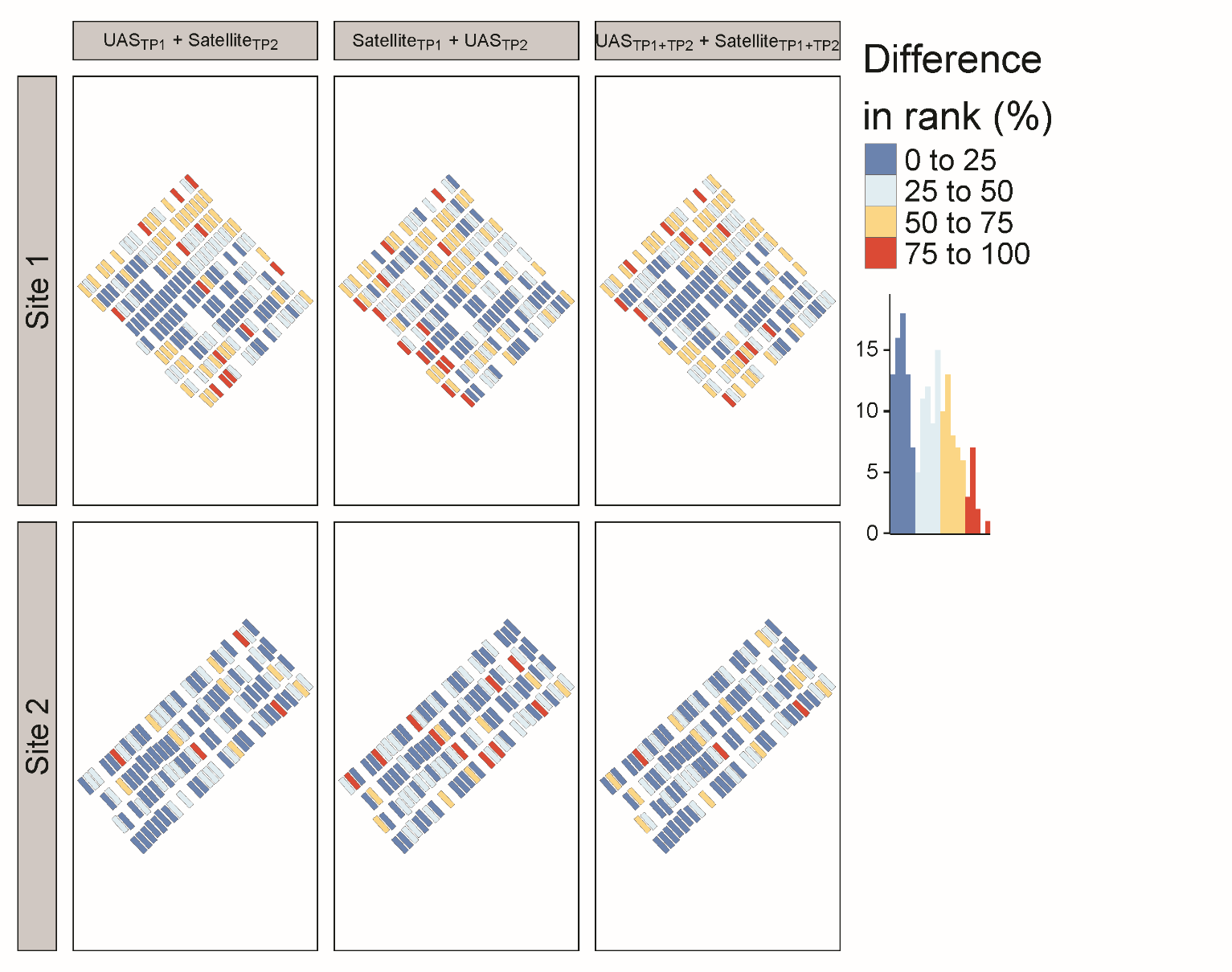


**Fig. 5.** Difference in relative ranking between actual and predicted seed yield using random forest models with multi-scale input spectral features (overall model performance presented in Table 2 in the manuscript).


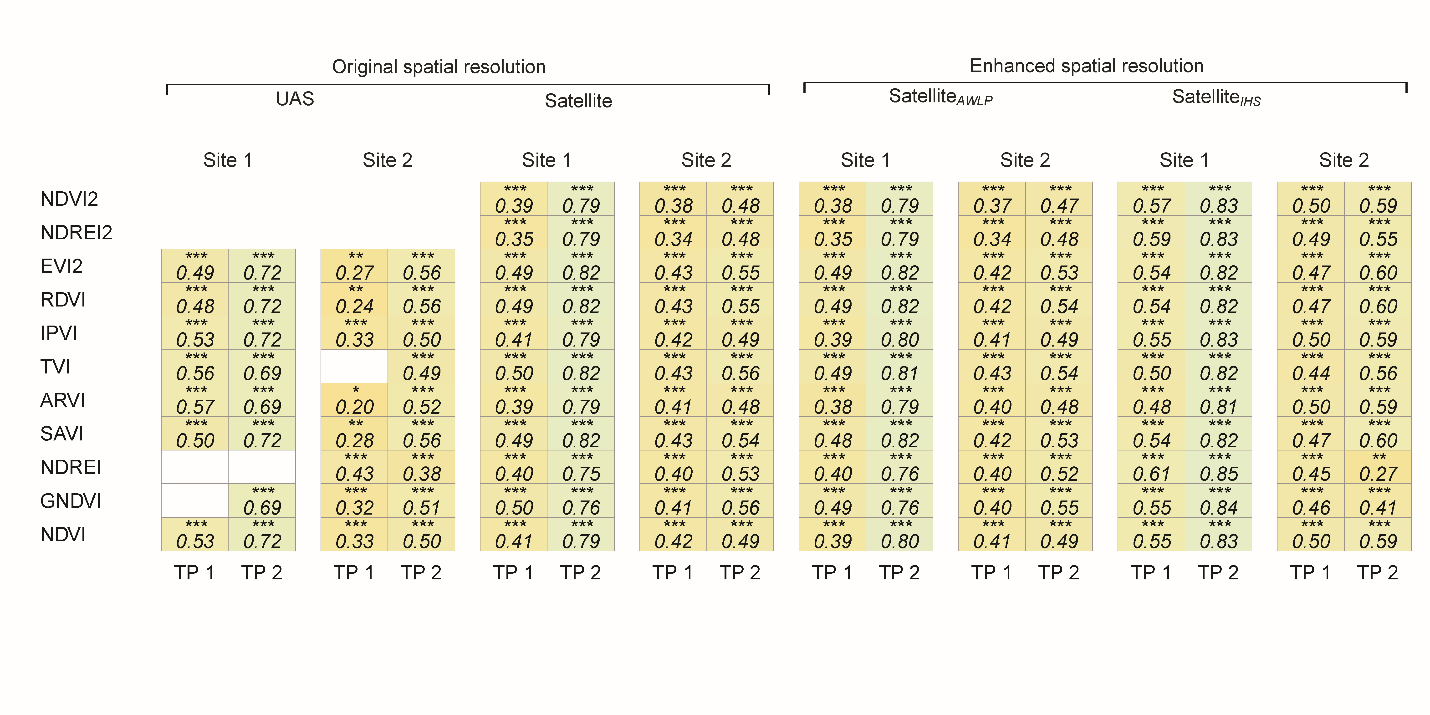


**Fig. 6.** Correlation coefficient between spectral features extracted from pan-sharpened satellite imagery and seed yield data using simulated UAS-panchromatic band at original spatial resolutions (at 0.02 m/pixel for Site 1 and at 0.03 m/pixel for Site 2). White cells indicate non-significant correlation (p ≥ 0.05), * (0.01 < p ≤ 0.05), ** (0.001 < p ≤ 0.01), and *** (p ≤ 0.001).


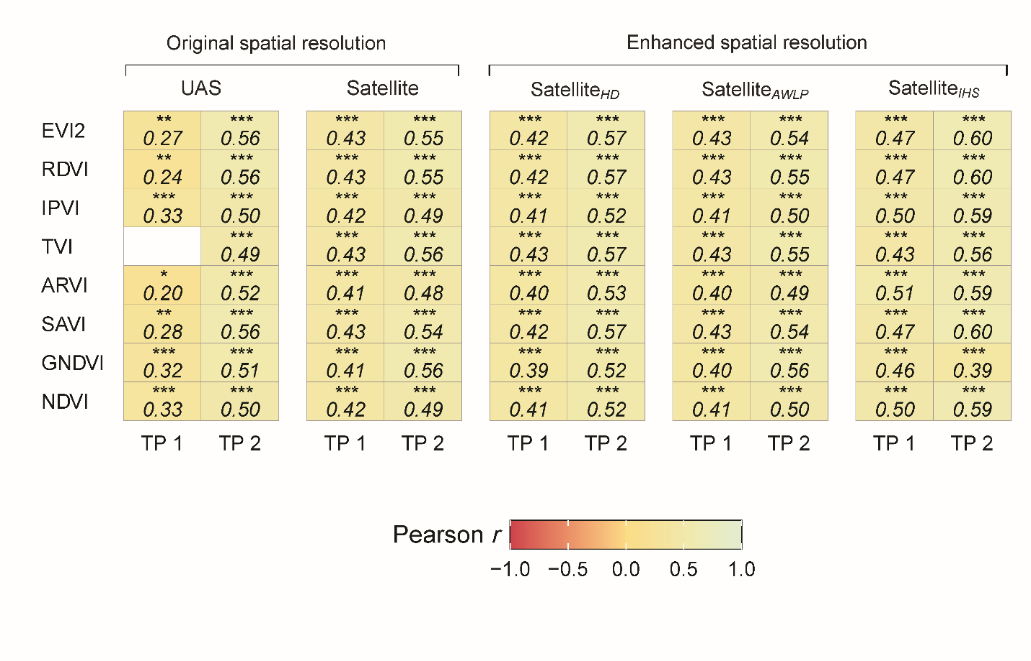


**Fig. 7.** Correlation coefficient between spectral features extracted from pan-sharpened satellite imagery (using simulated UAS-panchromatic band at 0.15 m/pixel) and HD Worldview imagery at 0.15 m/pixel with seed yield data. White cells indicate non-significant correlation (p ≥ 0.05), * (0.01 < p ≤ 0.05), ** (0.001 < p ≤ 0.01), and *** (p ≤ 0.001).


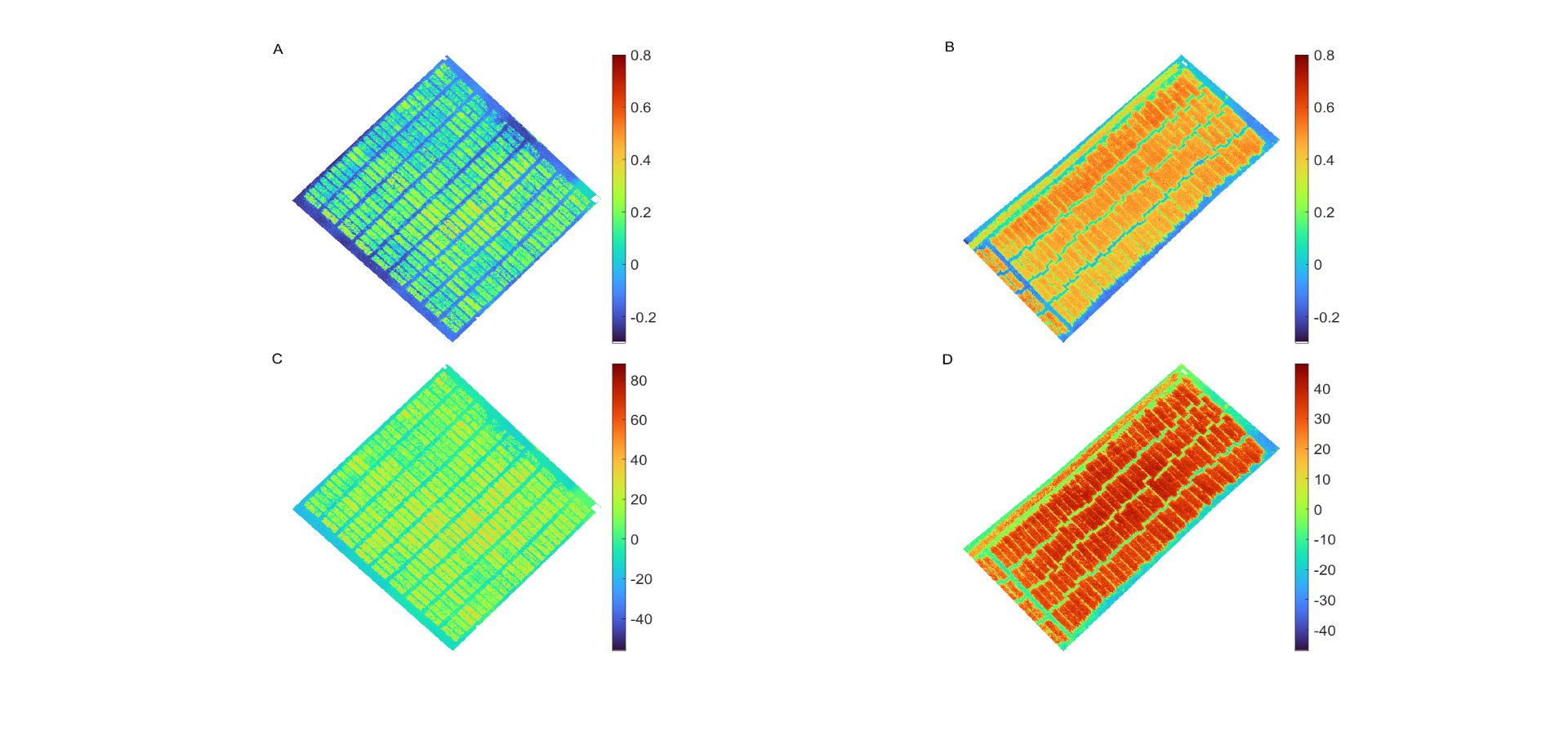

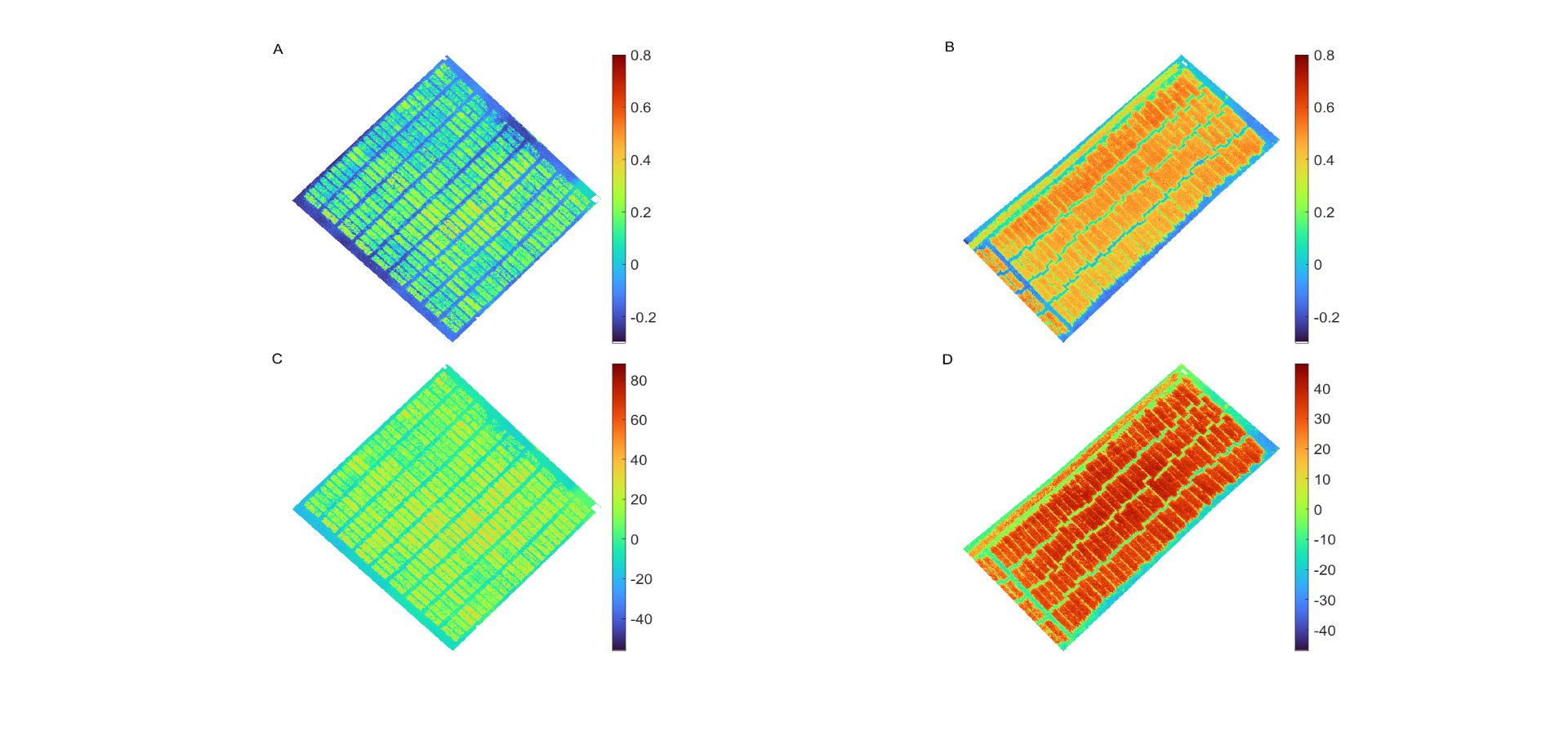


**Fig. 8.**  Green normalized difference vegetation index (A, B) and transformed triangular vegetation index (C, D) images extracted from UAS data acquired at TP 2 from Site 1 (A, C) and Site 2 (B, D).
